# Proto-SLIPS: Slippery Liquid-Infused Surfaces that Release Highly Water-Soluble Agents

**DOI:** 10.1021/acsami.5c09975

**Published:** 2025-09-09

**Authors:** Fengrui Wang, Jordan T. York, Takuma N. Kawamura, Douglas H. Chang, Sean P. Palecek, Helen E. Blackwell, David M. Lynn

**Affiliations:** 1 Department of Chemistry, 5228 University of Wisconsin−Madison, 1101 University Ave., Madison, Wisconsin 53706, United States; 2 Department of Chemical and Biological Engineering, 5228 University of Wisconsin−Madison, 1415 Engineering Dr., Madison, Wisconsin 53706, United States

**Keywords:** slippery surfaces, controlled release, drug delivery, antibiofouling, bacteria

## Abstract

Slippery liquid-infused porous surfaces (or “SLIPS”) can prevent bacterial surface fouling, but they do not inherently possess the means to kill bacteria or reduce cell loads in surrounding media. Past reports show that the infused liquids in these materials can be leveraged to load and release antimicrobial agents, but these approaches are generally limited to the use of hydrophobic agents that are soluble in the infused oily phases. Here, we report the design of so-called “proto-SLIPS” that address this limitation and permit the release of highly water-soluble (or oil-insoluble) agents. This approach involves the physical patterning of small, dried spots of hydrophilic drugs on the surfaces of hydrophobic porous materials and leads to analogs of conventional SLIPS that contain drug-patterned regions that can dissolve and disperse on exposure to water. We show that proto-SLIPS fabricated by patterning the antibiotic gentamicin on model porous PTFE membranes release drug rapidly, followed by a rapid process of self-healing in which oil from surrounding areas is transported to regions vacated by the drug. This healing process leads to uniform oil-infused surfaces with inherent antibiofouling properties similar to those of conventional SLIPS. The results of microbiological studies demonstrate that gentamicin-patterned proto-SLIPS can kill the common human bacterial pathogen *Staphylococcus aureus* on the surfaces of hydrogels or in liquid culture media and then transform to substantially reduce further bacterial surface fouling. This approach is modular and has the potential to enable the design of slippery surfaces that can release a wide variety of highly water-soluble and/or oil-soluble agents. In support of this goal, we demonstrate bases for the design of proto-SLIPS that release antifungal peptides, new dual-release coatings that release two agents targeted against different bacterial species, and the integration of concepts from the field of controlled release that provide additional measures of control over drug loading and release. We conclude that this proto-SLIPS strategy presents a new and useful approach to the design of drug-eluting SLIPS, with the potential to improve inherent antibiofouling behaviors and open the door to new applications of liquid-infused coatings in healthcare and other areas.

## Graphical abstract

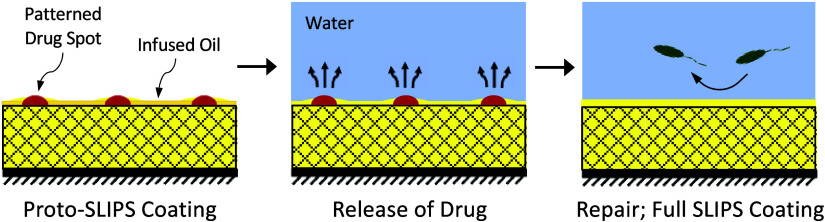

## Introduction

Contamination of surfaces by bacteria and other microorganisms can lead to substantial negative impacts in many healthcare contexts, ranging from the fouling or contamination of instruments and equipment to pain, suffering, and loss of life in patients, often due to infections on or around interventional devices and other implantable materials.^1–3^ Many different materials design strategies have been used to develop coatings that can mitigate bacterial biofouling and infection. While many of these strategies have shown promise and are effective on short time scales, they all ultimately fail when deployed in real-world scenarios or after use for extended periods.^1,2,4,5^ Effective, robust, and practical approaches that can reduce or prevent bacterial fouling and combat virulence in ways that move beyond conventional materials design strategies are urgently needed.

The work reported here was motivated broadly by past reports on the design of ‘slippery’ liquid-infused porous surfaces (or SLIPS). SLIPS comprise an emerging and promising class of synthetic coatings that exhibit robust antifouling properties, including the ability to substantially reduce surface colonization by bacteria.^6–15^ SLIPS are typically fabricated by the infusion of a lubricating liquid, usually a hydrophobic oil, into a porous surface to yield coatings that allow other liquids and contaminants, including microorganisms, mammalian cells, and other biological foulants, to simply slide (or ‘slip’) off.^9,12,14,16–21^ In the past decade, SLIPS and other liquid-impregnated surfaces (LIS) have become a substantial focus of research,^14,15,22–24^ and leading designs have been commercialized to prevent fouling in consumer and industrial environments.

Many SLIPS and other LIS are effective at repelling substances with which they come into direct contact^12,15,23–25^ and can thereby reduce contamination and colonization by bacteria and other cells.^6–15^ However, SLIPS are generally ‘passive’ materials and can do little to kill or attenuate the behaviors of organisms with which they *do not* come into direct contact, such as other nonadherent, or planktonic, bacteria in their environment. This is not an overall limitation if the goal is simply to keep a surface clean,^12^ but it can be problematic (and potentially fatal) if, for example, the surface is an implanted interventional device. As a class of materials, SLIPS are thus well equipped to prevent surface contamination but are poorly equipped to reduce bacterial load. It is also important to note that while many SLIPS are robustly antibiofouling, they are not perfect materials and can fail under certain conditions (e.g., under flow,^15,26^ which can cause infused liquids to drain and expose surfaces to which bacteria can subsequently adhere).^17^

One approach to the design of SLIPS that can affect changes in *nonadherent* microorganisms is to create liquid-infused surfaces that can release antimicrobial agents. Despite the conceptual simplicity of this approach, incorporation of release strategies into SLIPS has proven challenging, in part because of constraints imposed by the nature of the hydrophobic liquids that are used to design them and, from which, they derive their slippery properties. Many bioactive agents are insoluble in these infused oils; as a result, only a handful of past studies have reported strategies for the design of ‘drug-eluting’ SLIPS.^20,21,27,28^ Our group reported the first such system,^20^ demonstrating that the hydrophobic antimicrobial agent triclosan can be slowly released from SLIPS infused with silicone oil. That approach led to SLIPS that can prevent surface fouling by both fungal and bacterial cells (through inherent slippery character) and also kill pathogens and attenuate cell loads in surrounding media (by elution of triclosan).^20^ A key result of that study was the finding that the release of triclosan—and associated reductions in cell load—can also significantly improve inherent antibiofouling properties, even under conditions, such as exposure to flow, that may lead to loss of infused liquid and erosion of inherent antifouling behavior.^20^

Our prior work on drug-eluting SLIPS was enabled by the fact that triclosan is hydrophobic and, thus, soluble in the oily phases used to design SLIPS. This strategy is useful, but it is inherently limited to use with hydrophobic agents^20,21^ and thus precludes use of many other potent prophylactic and therapeutic actives—including many front-line small-molecule, peptidic, and carbohydrate-based antibiotics—that are exclusively soluble in water (or poorly soluble in oil) and could be exploited to (i) further improve antibiofouling properties and (ii) open the door to innovative new applications of slippery surfaces in healthcare contexts.

We recently reported that infusion of water-in-oil (w/o) nanoemulsions can be used to generate SLIPS and that the fugitive water droplets in these slippery two-phase liquids can be leveraged to host and sustain the release of water-soluble cargo.^29^ During the course of that work, we made the unexpected discovery that the infusion of conventional oils into porous polymer coatings that were physically patterned with small spots of the water-soluble antibiotic gentamicin can yield drug-patterned and slippery oil-infused coatings (which we refer to here as ‘proto-SLIPS’) that (i) release gentamicin when exposed to aqueous environments and, subsequently, (ii) transform or self-heal into coatings that are otherwise uniformly slippery and strongly antifouling.

In this report, we outline the design, fabrication, and characterization of this ‘proto-SLIPS’ approach, highlight key design parameters that influence the rapid or controlled release of patterned water-soluble (or oil-insoluble) agents and govern subsequent self-healing, and demonstrate the potential utility of this approach to design slippery, oil-infused coatings that can both substantially reduce surface biofouling by common microbial pathogens (via inherent slippery character) *and* reduce bacterial load in surrounding environments (by release of a clinically useful and highly water-soluble antibiotic). This proto-SLIPS approach is conceptually straightforward, modular in scope, and can be practiced using a variety of porous polymer substrates and patterned active agents. We demonstrate that, in addition to water-soluble small molecules, proto-SLIPS also can be generated by the physical patterning of charged antifungal peptides and degradable polymer microparticles, which provide additional avenues to target other microorganisms (e.g., fungal pathogens) and promote the sustained release of other active agents, respectively. This modular approach could also be used to mix and match the patterning of different types of agents to design proto-SLIPS that target multiple different species or types of microorganisms. Overall, this drug-patterned, ‘proto-SLIPS’ approach creates new opportunities to host and control the release of highly water-soluble active agents in ways that could advance the application of SLIPS and other LIS in healthcare contexts and in other commercial and industrial applications where fouling by microorganisms is problematic.

## Materials and Methods

### Materials

2-Vinyl-4,4-dimethyl azlactone (VDMA) was a kind gift from Dr. Steven M. Heilmann (3M Corp., Minneapolis, MN). Poly­(2-vinyl-4,4-dimethyl azlactone) (PVDMA, MW ≈ 87,000) was synthesized by free-radical polymerization of VDMA as described previously.^30–32^ Branched poly­(ethylenimine) (BPEI, MW ≈ 25 000), *n*-decylamine (95%), acetone (HPLC grade), tetrahydrofuran (THF, HPLC grade), dichloromethane (DCM, HPLC grade), polycaprolactone (PCL, MW ≈ 80,000), polyethylene glycol (PEG, Mw = 8,000), triclosan, poly­(d,l-lactide-*co*-glycolide) (PLG; lactide:glycolide, 50:50; 30–60 kDa), crystal violet (CV), gentamicin sulfate, and silicone oil (ρ = 0.963 g/mL, η = 45–55 cSt) were purchased from Sigma-Aldrich (Milwaukee, WI). Ethanol (EtOH, 200 proof) was obtained from Decon Laboratories (King of Prussia, PA). Food dye (McCormick assorted food colors) was purchased from Pick ‘n Save (Madison, WI). Unlaminated PTFE membrane filters (pore size = 0.2 μm, thickness ∼ 35 μm) were purchased from Sterlitech Corporation (Kent, WA). Glass microscope slides and polyethylene tubing (PE, outer diameter = 0.25 in.) were purchased from Fisher Scientific (Pittsburgh, PA). PLG microparticles were fabricated using previously reported methods.^33^ A model antifungal α,β-peptide (G-βL-F-βK–I–I-βK–K-βI-A-βK–S–F-NH_2_) was synthesized using previously reported methods.^34^

For bacteriological experiments, BacTiter-Glo microbial cell viability assay kits were purchased from Promega Corporation (Fitchburg, WI). Brain heart infusion (BHI) broth medium was purchased from Teknova (Hollister, CA). Lennox L Broth (LB) medium was purchased from Research Products International (Mt. Prospect, IL). Tryptic soy broth (TSB) medium was purchased from MilliporeSigma (Darmstadt, Germany). Water (18 MΩ) was purified using an Arium pro ultrapure water system (Sartorius). Fetal bovine serum (FBS) was purchased from Peak Serum (Wellington, CO). Gibco brand RPMI 1640 powder (containing phenol red and l-glutamine and without sodium bicarbonate or HEPES) and yeast peptone were purchased from Thermo Fisher Scientific (Waltham, MA). 3-(*N*-Morpholino)­propanesulfonic acid (MOPS) was purchased from Fisher Scientific (Pittsburgh, PA).

### General Considerations and Microbial Strain Information

Digital photographs and videos of agar plates and well plates were acquired using an iPhone XR smartphone. Pictures used to characterize the evaporation of aqueous droplets were acquired using a wireless pocket hand-held USB microscope (IWOBAC). Contacting footprints and sliding time data were analyzed using Microsoft PowerPoint 2021. All data were plotted using GraphPad Prism (version 7.0). Freezer stocks of *Staphylococcus aureus* (MW2) were maintained in 1:1 BHI:glycerol (50% v/v in Milli-Q water) and stocks of *Pseudomonas aeruginosa* (PAO1) were maintained in 1:1 LB:glycerol (50% v/v in Milli-Q water) at −80 °C. *Candida albicans* (SC 5314) purchased from ATCC (Manassas, VA) were maintained in 1:1 yeast peptone dextrose:glycerol stocks at −80 °C.

### Fabrication and Characterization of Proto-SLIPS

Porous polymer membranes were cut into desired sizes and placed onto acetone-washed glass microscope slides. The membranes supported on glass slides were then chilled briefly by placement on a bed of crushed dry ice for times ranging from a few seconds to 1 min depending on the type of therapeutic agent and the solution concentration, and then removed and placed on a flat surface at room temperature prior to drug patterning. We did not observe condensation of water onto the PTFE surfaces during these steps. This procedure was used in these proof of concept studies to promote the pinning of small water droplets on these superhydrophobic PTFE surfaces and thereby facilitate the placement of small, stationary aqueous droplets in desired positions. Desired volumes of aqueous solutions containing specified concentrations of active agent (see text) were then pipetted manually onto the surfaces, yielding surfaces hosting desired patterns of beaded aqueous droplets. These droplets were then allowed to evaporate under ambient conditions to yield hydrophobic surfaces physically patterned with desired patterns of dried spots (or dots) of active agent. Proto-SLIPS were then prepared by depositing a lubricating liquid (silicone oil) on the top surface of the PTFE membrane using a pipet. Samples were allowed to stand for several minutes to allow the liquid to infuse into the porous membrane (evident by a visual change in the opacity of the membrane) through capillary wicking. The excess liquid was then removed from the surface by dabbing with weighing paper. Conventional SLIPS not patterned with active agent were fabricated using these same general oil-infusion methods.

For the fabrication of proto-SLIPS using PVDMA/PEI-based coatings, the coatings were first fabricated on glass slides as previously described^20,31,35^ and then patterned and infused with oil using the same general methods described above. For proto-SLIPS designed for some dual-release applications (see text), triclosan was loaded into the porous polymer matrix prior to the patterning of the hydrophilic active agents. Loading of triclosan was achieved by treating the surfaces with a desired number of droplets (10 μL) of a triclosan solution in acetone (50 mg/mL). The acetone was then allowed to evaporate under ambient conditions for 5 min and hydrophilic active agents were then patterned on these surfaces using the methods described above. All samples were then dried under vacuum and infused with silicone oil using the general process described above.

Experiments to characterize droplet sliding times were performed in the following general manner. Briefly, oil-infused surfaces were mounted on a glass platform secured to the arm of a digital protractor, the protractor was set to a 5° angle, and a droplet of water of a defined and desired volume (e.g., 50 μL) was placed on the surface using a pipet. The time required for the droplet to slide down the surface over a fixed distance was recorded using a digital timer and used to calculate droplet sliding speeds. All measurements used to characterize droplet sliding speeds were performed in triplicate.

### Microbial Zone-of-Inhibition (ZOI) Assays

For bacterial ZOI assays, overnight cultures of *S. aureus* were grown in BHI at 37 °C with shaking at 200 rpm (until reaching an OD_600_ of approximately 1.5–2). Using a sterile swab, this overnight culture was spread evenly over the surface of a BHI agar plate. Substrates were then placed face-down on the coated agar and the plates were covered and incubated at 37 °C for 24 h. At the end of the 24-h period, the agar plates were photographed.

For antifungal ZOI experiments, *C. albicans* was streaked on a yeast peptone dextrose (YPD) agar plate from a frozen stock solution and grown overnight at 30 °C. For each assay, a colony was collected from the YPD plate and grown overnight in autoclaved test tubes at 30 °C with shaking in liquid YPD broth. Cells were then washed, resuspended in RPMI 1640 adjusted to pH 7.4 with 0.165 M MOPS, and prepared for subsequent experiments as described previously.^36^ The ZOI assay was then conducted according to the recommended CLSI guidelines.^37^ Cells were resuspended at a concentration of 10^7^ cells/mL in RPMI 1640 medium with MOPS, and a sterile cotton swab was dipped and streaked uniformly on the YPD agar plate three times to form a lawn. Proto-SLIPS samples loaded with antimicrobial peptides, control SLIPS samples, and bare glass substrates were placed onto the plates before a 24-h incubation at 30 °C.

### Well-Based Antimicrobial Assays

For well-based antibacterial assays, overnight cultures of bacteria were grown in BHI (*S. aureus*) or LB medium (*P. aeruginosa*) at 37 °C with shaking at 200 rpm. *S. aureus* subcultures were prepared by diluting overnight cultures 1:50 into fresh BHI medium. *P. aeruginosa* subcultures were prepared by diluting overnight cultures 1:50 into fresh LB medium. Substrates (agent-patterned proto-SLIPS, conventional non-patterned SLIPS, and bare glass) were incubated with either *S. aureus* or *P. aeruginosa* subcultures in a 24-well plate at 37 °C with shaking at 200 rpm. At 24 h, cell viability was characterized by transferring three 100 μL aliquots from each well to a clear-bottomed white 96-well microtiter plate. These aliquots were then mixed with 100 μL of BacTiter-Glo, prepared as described by the manufacturer’s protocol and then diluted 2x in Mili-Q water. The samples were allowed to sit in the dark for 5 min before measuring luminescence with a Synergy 2 plate reader (Biotek) running Gen5 1.05 software. For antifungal well-based experiments, 750 μL of 10^6^ cells/mL *C. albicans* subculture (5% fetal bovine serum in RPMI 1640 medium with MOPS) was added to each well in a 24-well plate containing samples and incubated at 37 °C without shaking for 24 h. A well with cells only and a well with media only were used as positive and negative controls, respectively.

### Crystal Violet (CV) Staining Protocol

The CV staining procedure was adapted from a previous report.^38^ Overnight cultures of bacteria were grown in TSB medium at 37 °C with shaking at 200 rpm. Subcultures were prepared by diluting overnight cultures 1:100 into fresh TSB medium and supplemented with glucose (0.05%). Substrates were incubated with subcultures in a 24-well plate statically at 37 °C. After 24 h, the substrates and controls were removed from their wells using forceps, gently washed once with PBS, and placed in the wells of a fresh 24-well plate. The samples were dried at 55 °C for 1 h before being stained via treatment with a 0.1% CV solution (weight/volume) for 5 min. Samples were then washed twice with Mili-Q water before imaging.

## Results and Discussion

Figure 1 shows a schematic representation and an overall physical picture of our drug-patterning ‘proto-SLIPS’ approach and the salient features that underpin this design. We reasoned that small aqueous droplets containing a water-soluble drug that were applied to hydrophobic porous polymer coatings (Figure 1A) and then allowed to evaporate (Figure 1B) would yield small solid ‘spots’ of drug that would remain surface-bound after the infusion of oil (Figure 1C), but dissolve readily upon exposure to aqueous environments (Figure 1D) and disperse drug into the aqueous phase at concentrations sufficient for bioactivity. This overall design leads to ‘proto-SLIPS’, or analogs of conventional SLIPS that contain small and locally ‘sticky’ drug-patterned regions that are not oil-infused but could, if designed strategically, be infiltrated or ‘backfilled’ by oil from surrounding areas as the drug is released (Figure 1E) and, thereby, transform or self-heal into uniformly oil-infused surfaces with inherent, long-lasting antifouling and antibiofouling properties similar to those of conventional SLIPS (Figure 1F). This approach is conceptually simple and has the potential to lead to slippery coatings that can strongly prevent surface biofouling (via inherent slippery character) *and* reduce microbial load (by release of antimicrobial agents). This approach also has the potential to be general, tunable, and modular, both in terms of the different classes of agents that can be exploited and the range of polymer coatings and biocompatible oils that can be used to design them. The sections below demonstrate the feasibility of this approach, identify key design parameters, and explore the breadth of agents, materials, and release profiles that can be exploited using this approach.

**Figure 1:**
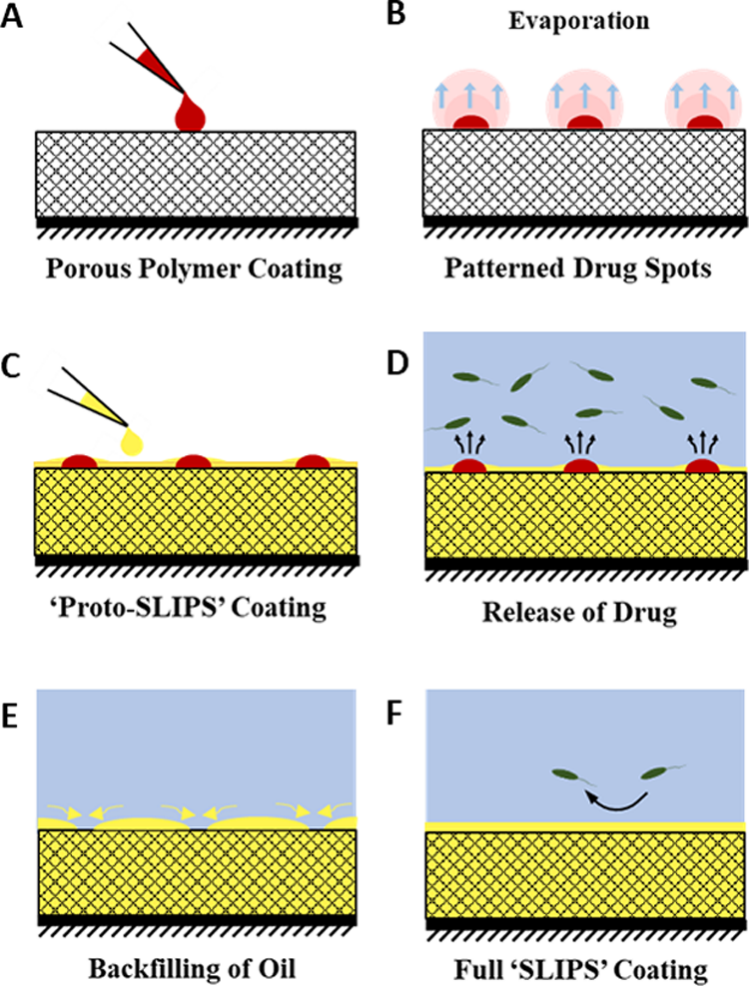
Schematic representation of our proto-SLIPS approach. Panels A-C illustrate steps associated with the fabrication of proto-SLIPS; panels D-F provide conceptualized schematic representations of key processes and behaviors of proto-SLIPS once they are submerged in aqueous environments, including bacterial cultures. (A) Aqueous droplets containing a therapeutic agent (red) are deposited or patterned onto the surface of a porous hydrophobic matrix (white). (B) The droplets deposited in (A) are allowed to evaporate, resulting in the formation of more compact, solid drug spots (depicted in darker red). (C) The drug-patterned porous surfaces are then infused with a hydrophobic oil (yellow), resulting in proto-SLIPS, or oil-infused, slippery surfaces that are patterned with drug spots that are not themselves oil-infused. (D) After placing the samples into an aqueous environment (blue; planktonic bacteria are indicated in green), the hydrophilic drug dissolves into the aqueous media. (E) After the release of the drug, which can result in the killing of planktonic bacteria, small nonoil-infused regions in areas vacated by the removal of drug are backfilled by the transport (represented by yellow arrows) of infused oil from other regions. (F) Backfilling of oil results in a uniform and continuously oil-infused surface with characteristics similar to those of conventional SLIPS, including the ability to prevent surface colonization by additional planktonic bacteria (represented schematically and shown in green).

### Fabrication and Characterization of Proto-SLIPS Patterned with Gentamicin

The first step in the design of proto-SLIPS involves the patterning of an underlying porous polymer matrix with small hydrophilic spots or ‘dots’ of a highly hydrophilic (and oil-insoluble) agent by the placement and evaporation of small droplets of aqueous solutions (Figure 1A-B; see Materials and Methods for details). In this study, we used (i) aqueous droplets of the water-soluble, broad-spectrum antibiotic gentamicin deposited manually via micropipette and (ii) hydrophobic microporous PTFE membranes used in past studies for the design of conventional SLIPS.^6,13,19,20,24^ In general, this process exploits the hydrophobic nature of the PTFE membranes, which are not readily wet by water, to create concentrated dots of gentamicin confined primarily at the topmost surface of the membrane, as shown in Figure 1B. To explore the feasibility of this approach, we first performed a series of experiments to characterize the impacts of droplet volume and gentamicin concentration on the formation of gentamicin spots.

We placed droplets of varying volumes and concentrations of gentamicin on PTFE membranes and characterized the droplet evaporation process by tracking the change in the transient contact radius of the droplets over time. Figure 2A shows the time-based evolution of the nondimensional wetted radius for 5 μL droplets of either a 10 mg/mL (closed squares) or a 100 mg/mL (closed circles) gentamicin solution evaporating on the PTFE surface at ambient temperature. These droplets exhibited two distinct evaporation patterns. The 100 mg/mL droplets maintained a constant contact radius (CCR) during evaporation, indicating minimal changes in their wetted footprint.^39–44^ This behavior is consistent with the fact that this concentration is near the reported solubility limit of gentamicin in water (110 mg/mL), likely resulting in the precipitation of gentamicin along the contact line early in the evaporation process. In contrast, 10 mg/mL droplets initially followed CCR-type behavior but then exhibited a significant decrease in transient contact radius (Figure 2A, closed squares), maintaining a constant contact angle (CCA) during most of the evaporation before returning again to CCR-type behavior.^41–44^ This shift toward a CCA mode early in evaporation, followed by a return to a CCR mode, is consistent with findings reported for the evaporation of aqueous solutions on other types of superhydrophobic surfaces.^39–43^ Figure 2B shows a series of overlaid images at various time points during the evaporation of 5 μL droplets of 10 and 100 mg/mL gentamicin solutions. These images further illustrate these concentration-dependent differences in evaporation as well as the notable reduction in the footprint of the 10 mg/mL droplet, as compared to the 100 mg/mL droplet, as evaporation occurs.

**Figure 2:**
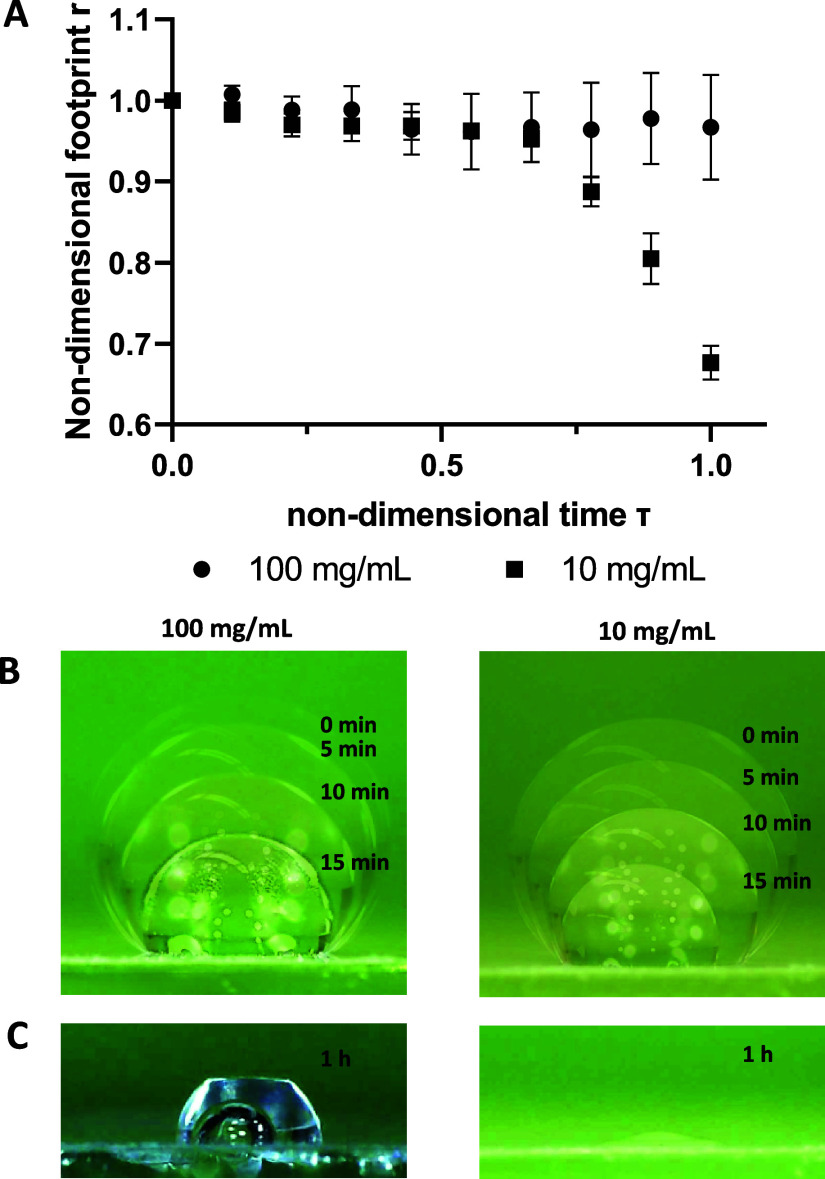
Characterization of the evaporation and drying of aqueous droplets containing gentamicin on PTFE membranes. (A) Plot showing the nondimensional wetted radii for 5 μL droplets of either a 10 mg/mL (closed squares) or a 100 mg/mL (closed circles) gentamicin solution evaporating over the first 45 min on PTFE surfaces at ambient temperature. Changes in footprints during the evaporation process were measured by collecting side-view images of evaporating droplets, similar to those shown in panels B–C, and quantified using Microsoft PowerPoint. Data were corrected based on initial droplet footprints and a decrease in number indicates a decrease in footprint over time. The error bars represent the standard deviation of three trials of the evaporation experiments. (B) Representative overlaid time-lapse images showing the droplet evaporation process for 5 μL droplets of 100 mg/mL (left) and 10 mg/mL (right) gentamicin in the first 15 min. (C) Representative images of the final evaporated drug dots resulting from the complete evaporation of the droplets shown in B acquired at the same camera angle, position, magnification, and scale. For reference, the dried drug dot shown in Figure 2C (left) arising from the evaporation of the 100 mg/mL solution is approximately 1.3 mm across with an estimated height of 0.6 mm and the dried drug dot shown in Figure 2C (right) arising from the evaporation of the 10 mg/mL solution is approximately 0.6 mm across with an estimated height of 0.08 mm.

It is also worth noting that, as these droplets evaporated, small air bubbles emerged at their aqueous-solid interfaces (see Video S1 of the Supporting Information for a representative video showing the evaporation process). For superhydrophobic surfaces in the Cassie–Baxter state, this phenomenon is often attributed to small pockets of air entrapped between the surface and a droplet; during droplet evaporation, these pockets of air are gradually released due to changes in interfacial tension.^39,42,44–46^ In this present work, air bubbles observed in 10 mg/mL droplets are released along the droplet edge as the contact line retracts incrementally (see Video S1), driven by local pinning and depinning from the surface. Conversely, in 100 mg/mL droplets, which are constrained by a pinned contact line that hinders transverse shrinkage, smaller pockets of air did not readily escape and, instead, generally accumulated to form a larger pocket of air that remained entrapped in the solid gentamicin spot after complete evaporation. Figure 2C shows images of two gentamicin spots created by the complete evaporation of a 10 mg/mL and a 100 mg/mL droplet and reveals large differences in the footprints and volumes of the spots. Further inspection also reveals larger and visible air bubbles, or voids, entrapped in spots created by the evaporation of the 100 mg/mL droplets. These internal voids may be advantageous with respect to the temporary storage of excess oil and, subsequently, the promotion of rapid self-healing in proto-SLIPS once their gentamicin spots are dissolved in water. We return to these observations again in the sections below.

We next conducted a series of experiments to characterize the influence of gentamicin-patterned drug spots on the infusion of silicone oil into PTFE membranes (e.g., as shown schematically in Figure 1C). The infusion of silicone oil into PTFE membranes results in a shift in the optical appearance of the membranes from white and opaque, in the absence of oil, to transparent, when fully infused, providing a straightforward way to assess rates and extents of infusion over time. Several interesting observations emerged from this analysis. First, although areas of the membrane directly under gentamicin spots required a longer time to become infused than the unpatterned regions surrounding them, they eventually turned visibly transparent, suggesting that oil was able to successfully penetrate areas underneath the spots (see Figure S1). Second, the amount of time to become fully infused correlated with the sizes of the gentamicin spots: smaller spots (e.g., a 5 μL droplet of 10 mg/mL solution) became fully translucent within minutes, while larger spots (e.g., a 10 μL droplet of 100 mg/mL solution) often required several hours. Third, we observed substantial differences in the dynamics of infusion in areas under gentamicin spots created using more concentrated 100 mg/mL solutions as compared to spots created by evaporation of less concentrated solutions. In general, areas directly underneath the large voids of air, nearer to the centers of these larger spots, became translucent first; in spots created using 10 mg/mL solutions, we generally observed infusion to begin at the perimeter of the spot and then gradually progress toward the center (see Figure S1). This behavior suggests differences in the distributions of oil in SLIPS patterned with gentamicin spots created using different concentrations and provides support for the possibility that large voids observed in spots created using more concentrated solutions could act as reservoirs for oil (a possibility that, as noted above, could influence rates and mechanisms of self-healing in these materials). Overall, these results suggest that the physical patterning of gentamicin spots does not adversely influence the infusion of lubricating oil in areas surrounding the spots or directly underneath these spots.

### Characterization of Slippery Behavior and Processes of Self-Healing after Contact with Water

We then characterized the mobilities and wetting behaviors of aqueous liquids placed on the surfaces of gentamicin-patterned proto-SLIPS. Initial experiments demonstrated that proto-SLIPS fabricated by the infusion of oil into gentamicin-patterned PTFE (i) exhibit slippery behaviors characteristic of conventional SLIPS in areas that are not drug-patterned and (ii) that patterned areas are not slippery but are instead substantially wetted by water. To further characterize slipperiness and changes in slippery behavior, we used an experimental setup similar to that depicted in Figure 3A to monitor the sliding behaviors of small droplets of water (50 μL) on proto-SLIPS held at a 5° tilt angle. Figure 3B shows representative images of a water droplet (containing green food dye; to guide the eye) sliding down the surface of a proto-SLIPS sample patterned with a single gentamicin spot formed by the evaporation of a 5 μL droplet of 100 mg/mL gentamicin solution (the location of the drug spot is indicated by the small red arrow in Figure 3B; this drug spot is also indicated schematically in Figure 3A, and is colored red to guide the eye and facilitate further discussion below).

**Figure 3:**
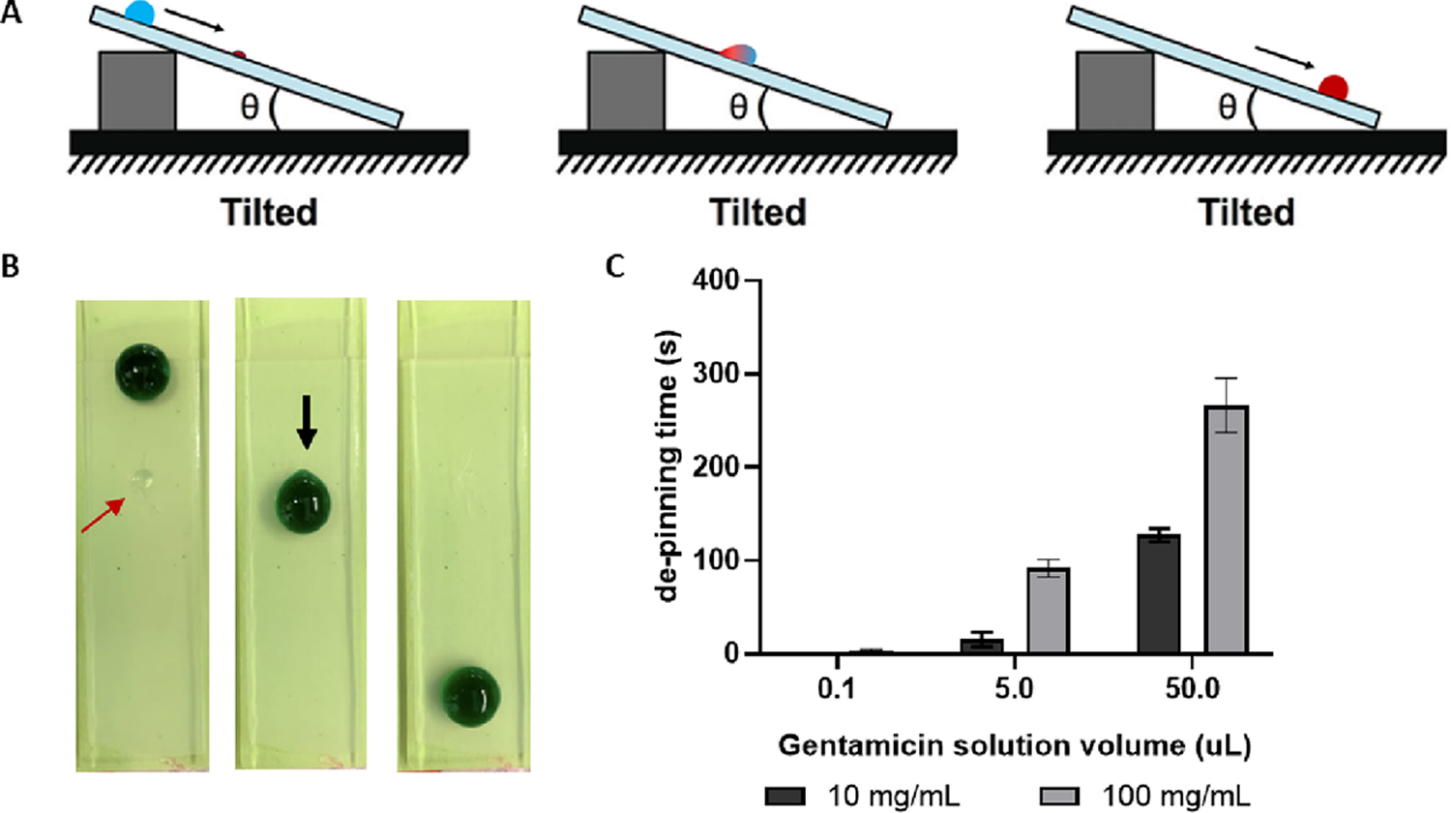
(A) Schematic representation of the experimental setup used to characterize surface slipperiness. Samples were placed at a 5° tilt angle and water droplets (50 μL; shown in blue in the image at left) were placed on them at the top (elevated) end. As water droplets slide down the surface of a proto-SLIPS and encounter a drug spot (shown in red), the water droplets pin in place and the hydrophilic gentamicin starts to dissolve into the contacting water droplet (depicted visually as a blue/red pinned droplet in the center image). Once the drug is fully released, the water droplet (now shown in red in the image at right) depins and continues to slide down the surface. (B) Top-down images of a water droplet (colored green using a food dye to guide the eye) sliding down the surface of a proto-SLIPS containing one gentamicin spot (position indicated by the red arrow) patterned by the evaporation of a 5 μL droplet of 100 mg/mL gentamicin. The black arrow in the center image shows the direction of the sliding droplet. The droplet pins at the drug dot (center panel) and regains motion again after 120 s (right panel). (C) Plot of the amount of time required for a water droplet (50 μL) to regain motion (denoted as the depinning time) after pinning at gentamicin spots created by varying gentamicin loading volumes and concentrations. The error bars represent the standard deviation of three trials of the depinning experiments for each drug loading condition.

In this experiment, the water droplet initially slid freely down the surface at a rate of approximately 0.12 cm/s (e.g., Figure 3B, left), but became pinned upon contact with the gentamicin spot, adopting a teardrop shape with different advancing and receding water contact angles (e.g., Figure 3B, center). However, after approximately 120 s, we observed the water droplet to continue sliding, unhindered and at a rate of approximately 0.10 cm/s, down the surface (e.g., Figure 3B, right). Interestingly, additional water droplets placed at the top of the substrate slid down unimpeded, at a rate of approximately 0.11 cm/s, over both the location of the patterned spot and the entire length of the membrane. We interpret this result to suggest two important outcomes related to the potential utility of this proto-SLIPS design: first, the water-soluble gentamicin contained in a patterned spot can be rapidly dissolved (e.g., in ∼ 2 min) into a pinned water droplet (as depicted schematically in Figure 3A) and, second, the effective removal of gentamicin from the proto-SLIPS surface appears to permit additional transport of oil in ways that can promote the transformation or self-healing of the surface to a uniform and continuous slippery surface that behaves in a manner characteristic of conventional SLIPS.

We conducted additional experiments using the general experimental setup described above to characterize the impacts of gentamicin solution loading volume and concentration on the proto-SLIPS recovery process. The results of these studies are summarized in Figure 3C and are expressed as differences in the amount of time required for pinned droplets to regain sliding mobility. Inspection of these results reveals that all tested conditions resulted in pinning of sliding water droplets and that all pinned droplets eventually regained mobility, indicating effective healing. Overall, the time required to regain mobility, taken here as an initial measure of recovery, was proportional to the loading volume of gentamicin. For drug spots created using droplets of the same volume, higher concentrations (100 mg/mL) resulted in larger footprints, as described above, and thus to longer recovery times compared to spots created at lower concentrations (see Table S1 for additional information on differences in areal footprints and the amount of time required for droplets to regain mobility for conditions tested in Figure 3C). We consider it likely that the recovery process requires the transport or redistribution of oil from other nearby locations. Our results suggest the presence of sufficient amounts of excess oil in these proto-SLIPS to accommodate and render slippery regions that are vacated by the dissolution of gentamicin spots. Because larger gentamicin spot footprints would likely require more time for this redistribution process to occur, the results shown in Figure 3C are consistent with this general physical picture.

To further investigate the potential impacts of substantially larger gentamicin spot sizes on healing and recovery processes, we designed a PTFE-based proto-SLIPS by depositing a 100 μL droplet of a 100 mg/mL aqueous gentamicin solution. Evaporation of this droplet resulted in a large solid gentamicin spot extending over approximately 40% of the width of the PTFE membrane, with a visible entrapped air pocket. In this case, the initial oil infusion process required several hours, and followed a visual pattern consistent with that discussed above for other large spots—we observed oil infusion to occur first around the outer circumference of the spot and in the entrapped air pocket, creating an inner concentric ring surrounding the void that was then infused more slowly (see Figure S2 A-C of the Supporting Information for details). After infusion and the removal of excess oil, we added water droplets to promote dissolution of gentamicin. Subsequent removal of the added water droplets revealed small glossy patches of oil on the SLIPS surface (see Figure S2 D; these patches were confirmed to be oil, and not residual water or gentamicin solution, using an oil-soluble dye). These patches of excess oil were generally observed to be positioned at or near the trapped pockets of air, and provide additional support for the proposition that the voids in these gentamicin spots can act as small, internal reservoirs of oil that can be redistributed after the dissolution of gentamicin and, in general, assist and promote healing and recovery—even in gentamicin spots as large as the one used in this experiment. The overall time required for healing in this extreme case extended to about 10 min. At that point, water droplets were able to slide freely over the area initially covered by this large gentamicin spot, consistent with a full functional recovery and the transformation of a proto-SLIPS to a more uniform conventional SLIPS surface. It is reasonable to anticipate that there are functional limits to the sizes and number of gentamicin spots that can be used to design proto-SLIPS that can fully heal in this way. Ultimately, the behaviors of these materials will be influenced by parameters such as loading concentration and the presence of voids, as discussed above, and other factors, such as spot density and infused membrane thickness, typically associated with SLIPS design.

### Gentamicin-Loaded Proto-SLIPS Reduce Bacterial Load and Improve Antibiofouling Behavior

We next conducted a series of experiments to characterize the ability of proto-SLIPS patterned with gentamicin spots to release gentamicin, reduce bacterial loads, and undergo healing and recovery when exposed to cultures of *S. aureus*, a clinically relevant opportunistic pathogen, growing either on the surfaces of agar gels or in liquid media. For these studies, we designed four proto-SLIPS patterned by the evaporation of different volumes of droplets of aqueous gentamicin (10 mg/mL). Five droplets were deposited on each chip, and we used droplet volumes ranging from 0.1 μL (resulting in ∼ 0.2 mm diameter spots covering ∼ 0.3% of the total membrane area) to 5 μL (yielding ∼ 0.6 mm diameter dots covering ∼ 1% of the total membrane area). Figure 4B shows a schematic illustration of relative spot-size and corresponding gentamicin loading differences in the set of five proto-SLIPS used in the microbiological experiments described here.

**Figure 4:**
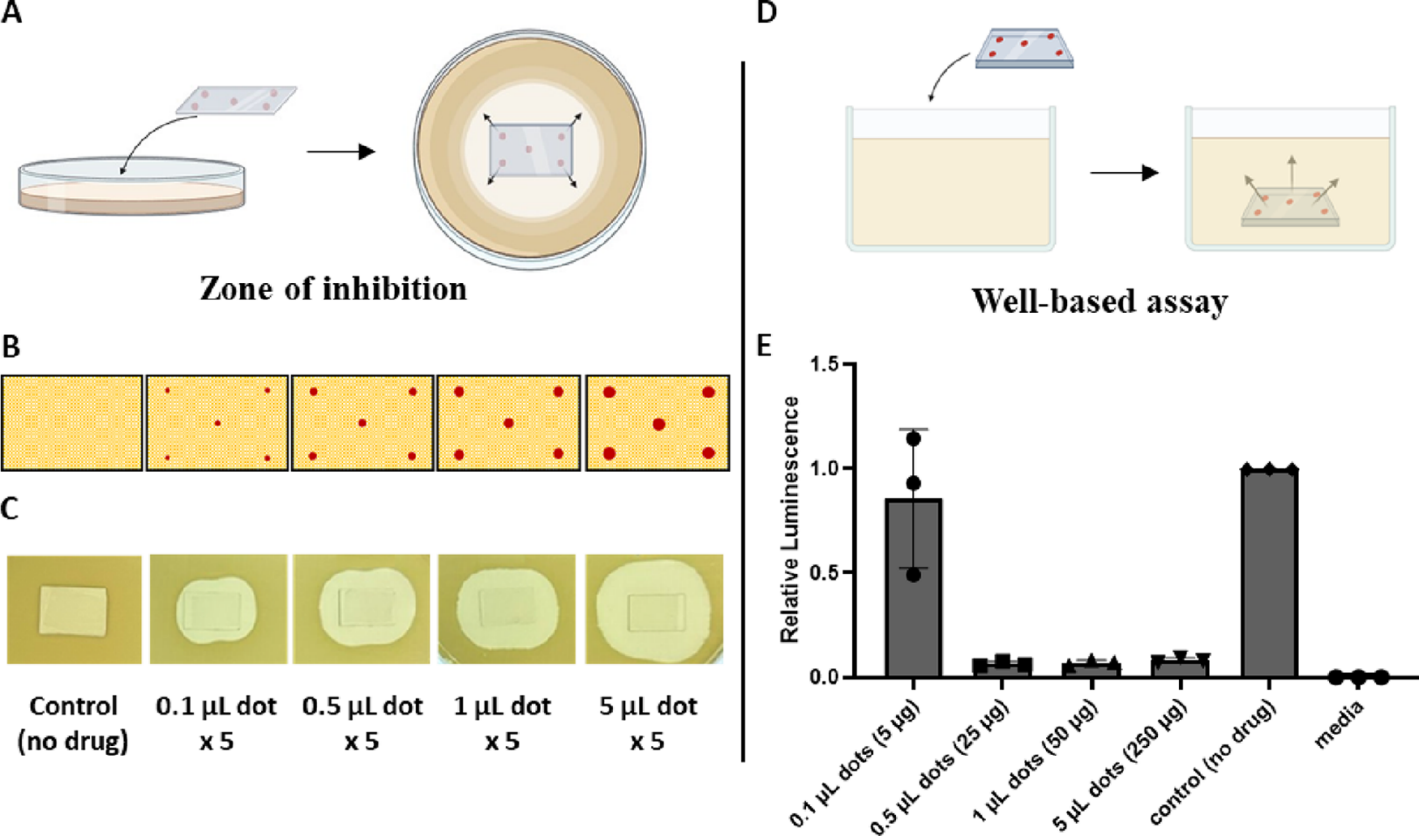
Characterization of gentamicin-patterned proto-SLIPS using antimicrobial assays. (A) Schematic representation showing a standard microbiological zone-of-inhibition (ZOI) assay. In this example, a proto-SLIPS surface is placed face-down onto the surface of an agar gel inoculated with bacteria (side-on view shown at left). After overnight incubation, visually clear zones surrounding the substrate indicate regions containing dead bacteria (top-down view shown at right; see text for additional details). (B) Schematic representations of proto-SLIPS made by depositing 0 or 5 droplets of a 10 mg/mL gentamicin solution at the corners and at the center of the PTFE membrane. Drug spots are represented by red circles and the yellow rectangles represent oil-infused PTFE membranes. Four different drug loading volumes were used (0.1 μL, 0.5 μL, 1 and 5 μL, from left to right). These schematic representations show relative differences in the areal footprints of the drug spots created using different loading volumes. (C) Representative results of ZOI assays showing bright zones characteristic of dead bacterial cells surrounding all proto-SLIPS after 24 h of incubation (the notation “x 5” indicates 5 spots per proto-SLIPS sample); conventional SLIPS that were not patterned with drug spots were used as controls (left) and did not lead to observable zones of inhibition. (D) Schematic illustration of well-based cell viability assays. Proto-SLIPS samples (analogous in size and patterning to those used in the experiments shown in panel C were immersed in cultures of bacteria for 24 h and cell viabilities were then quantified using a luminescence-based assay. (E) Results of well-based viability assays as a function of gentamicin spot size and loading. Lower luminescence values are associated with fewer live bacteria. The graph shows the average results of three independent cell viability assays, each with a triplicate of samples for all drug loading conditions. The error bars represent the standard deviation of the nine measurements.

We first characterized the ability of gentamicin-patterned proto-SLIPS to release gentamicin, kill *S. aureus*, and reduce microbial loads using a standard contact-mediated antimicrobial zone-of-inhibition (ZOI) assay. The overall design of this experiment, in which the release, diffusion, and activity of an antibiotic from a substrate placed on an agar gel can be characterized by the presence and size of a visible ring (or ‘zone’) containing dead bacteria surrounding the substrate, is shown in Figure 4A (see Materials and Methods for details). Figure 4C shows representative results of ZOI assays performed using the gentamicin-patterned proto-SLIPS described above and shown in Figure 4B. Inspection of these results reveals bright zones characteristic of dead bacterial cells around each substrate after 24 h of incubation (the darker gold regions surrounding those zones are areas of the agar gel containing live *S. aureus*). As a key control, conventional PTFE-based SLIPS that were not patterned with gentamicin did not result in any observable zones of inhibition (see Figure 4C). Further inspection of these results also reveals the ZOIs surrounding these substrates to vary in size with the sizes and loading concentrations of the patterned gentamicin spots, demonstrating that these design parameters can be used to tune the amount of gentamicin released and the resulting spatial area over which bacterial load can be reduced in this assay.

In a second series of experiments, we characterized the antimicrobial and antibiofouling behaviors of proto-SLIPS after direct immersion into cultures of *S. aureus* growing in liquid medium. For these experiments, proto-SLIPS were submerged in bacterial culture for 24 h (as shown schematically in Figure 4D) and cell viability was quantified using a BacTiter-Glo assay (see Materials and Methods). The results of these experiments are shown in Figure 4E. Levels of cell viability measured for proto-SLIPS patterned using the smallest drug loading volume (0.1 μL) were high and, in general, statistically similar to no-drug control SLIPS, suggesting that the total amount of gentamicin loaded and released on those surfaces was not sufficient to exceed the MIC of gentamicin in *S. aureus* under the conditions used here. Further inspection of Figure 4E, however, reveals that all other proto-SLIPS containing higher gentamicin loadings (ranging from 12.5 μg/mL to 125 μg/mL) were able to substantially reduce bacterial load, exhibiting very low levels of cell viability that were similar to those of no-bacteria controls.

Additional experiments demonstrated that the proto-SLIPS used above can fully self-heal and recover after the release of gentamicin in ZOI experiments or in liquid cultures and strongly reduce subsequent surface fouling by *S. aureus*. For these experiments, we used immersion into water containing a green dye as a visual indicator of self-healing. The conventional, unpatterned SLIPS failed to attract water either prior or after incubation in *S. aureus* cultures (Figure 5A). In contrast, prior to incubation with bacterial cultures, proto-SLIPS immersed into these same dye solutions collected small and readily visible green droplets of water in the locations of the patterned gentamicin spots (Figure 5B; the left image shows a representative example for a surface patterned with five gentamicin spots after immersion in the green dye). However, after incubation on agar or in cultures of *S. aureus* for 24 h, these samples were free of any visible green droplets of water after immersion into dye solution (Figure 5B, right), analogous to the parent SLIPS (Figure 5A, right). In addition, larger test droplets of water were able to slide unimpeded over the surfaces of these materials (similar to results shown in Figure 3 and discussed in the section above; not shown). (We note, for clarity, that the areas of green visible near the edges of the samples shown Figure 5A-B for both conventional SLIPS and proto-SLIPS arise from water adsorbed to the bare glass substrate on which the oil-infused PTFE is supported; the PTFE membranes were cut slightly smaller than the underlying glass substrate in these experiments.) Overall, these results indicate that proto-SLIPS exposed to bacterial cultures were able to then transform into uniform slippery surfaces that were visually and functionally indistinguishable from conventional, unpatterned SLIPS.

**Figure 5:**
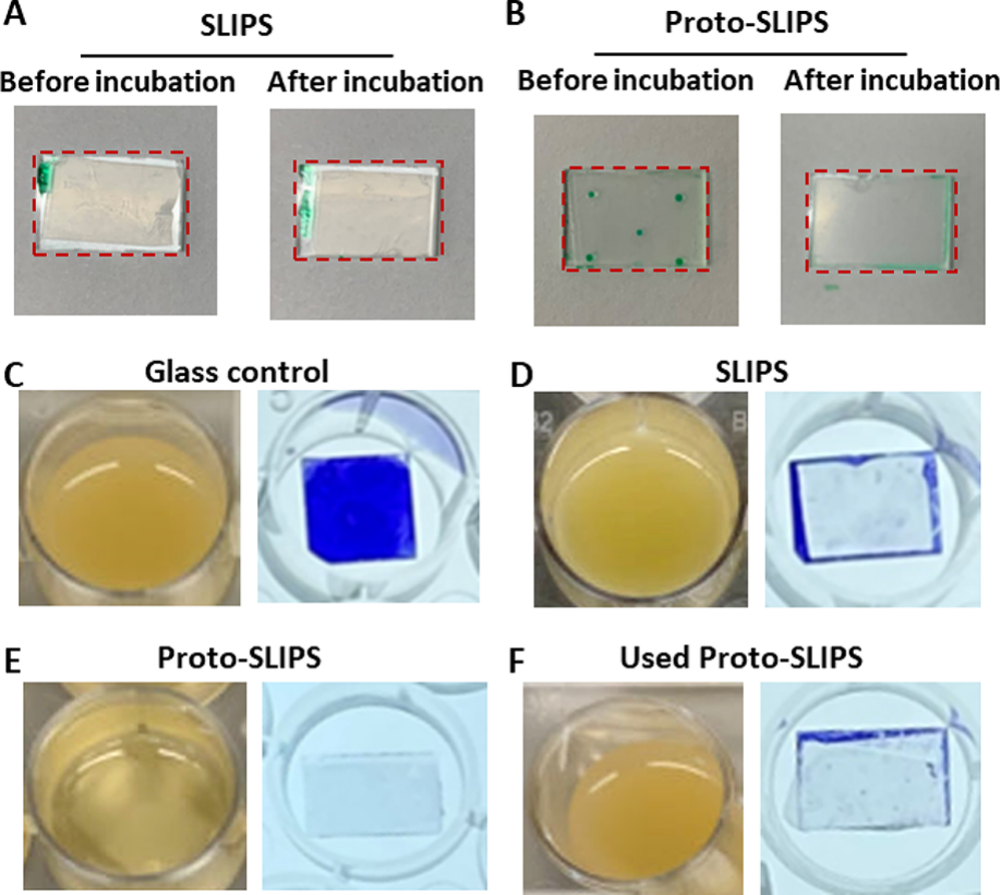
Results showing characterization of the extent of repair and self-healing of proto-SLIPS after use in microbiological assays. Representative examples shown here are for proto-SLIPS designed with five drug spots created by evaporating 1 μL droplets of a 10 mg/mL aqueous gentamicin solution. (A-B) Images of conventional, unpatterned SLIPS (A) and gentamicin-patterned proto-SLIPS (B) after submersion for two seconds in water containing a green dye both before (left) and after (right) overnight incubation with *S. aureus* in both well-based assays and ZOI assays (the representative images shown here are for samples used in ZOI assays). Areas that are not slippery can be visualized by the presence of green water adhered to the surface. Areas of green visible near the edges of the samples arise from water adsorbed to the bare glass substrate on which the oil-infused PTFE is supported; the PTFE membranes were cut slightly smaller than the underlying glass substrate in these experiments. (C–F) Representative results after the submersion and incubation of a bare glass control surface (C), a conventional, unpatterned SLIPS surface (D), a gentamicin-patterned proto-SLIPS surface (E), and a ‘used’ gentamicin-patterned proto-SLIPS surface (F; see text) in cultures of *S. aureus* followed by biofilm analysis using the CV staining method. The left side of each pair of images shows pictures of the wells in which the surfaces were incubated and shows differences in solution turbidity (tan and opaque appearance is an indicator of high growth; tan and transparent is an indicator of low growth). The right side of each pair shows images of each surface after removal of culture, washing, and CV staining; dark blue/purple color indicates the presence of adhered bacteria/biofilm.

Figure 5C–F shows the results of an experiment in which (i) a bare glass substrate, (ii) a conventional (not drug patterned) PTFE-based SLIPS surface, (iii) a fresh gentamicin-patterned proto-SLIPS surface, and (iv) a ‘used’ gentamicin-patterned proto-SLIPS surface that had been previously retrieved after 24 h of submersion in bacterial culture) were incubated for 24 h in *S. aureus* cultures under conditions that permit the formation of bacterial biofilm (or more generically, substantial surface biofouling). We then visually inspected the bacterial cultures and characterized the substrate surfaces by removal, washing, and staining with crystal violet (CV), to obtain visual indicators of differences in bacterial growth (evident via solution turbidity in each sample well) and surface biofouling (evident as regions that appear blue/purple after staining; see Materials and Methods for details). Inspection of the images in Figure 5C reveals a high degree of culture turbidity (panel C left), consistent with a high degree of bacterial growth, and dark and uniform CV staining (panel C, right) across the entire bare glass substrate. In contrast, Figure 5D, which shows results for a conventional SLIPS surface, shows high degrees of culture turbidity and low levels of CV staining; these results are consistent with the ability of conventional SLIPS to strongly reduce surface biofouling, but the inability of conventional SLIPS to kill planktonic bacteria. (We note again that areas of blue staining near the edges of the surface shown in the right panel of Figure 5D arise from the formation of bacterial biofilm on portions of the underlying bare glass substrates on which the liquid-infused PTFE membranes were supported; see also discussion above).

The results in Figure 5C–D contrast sharply to those shown in Figure 5E, which shows results for a gentamicin proto-SLIPS surface. In this case, these drug-eluting surfaces are able to (i) substantially reduce solution turbidity in the sample wells, consistent with a substantially reduced load of planktonic bacteria, and (ii) self-heal and strongly prevent biofouling on both the surface of the SLIPS and on areas near the edges of the underlying bare glass substrates on which they were supported. Finally, the images shown in Figure 5F show that ‘used’ proto-SLIPS that were allowed to completely release gentamicin and then heal prior to use in these experiments remain substantially free of bacterial colonization and yet, because they no longer contain gentamicin, do not substantially reduce bacterial loads in the surrounding culture (similar to the behavior of the conventional SLIPS surface shown in the right panel of Figure 5F). We note that, for clarity, all CV-stained images shown in Figure 5C–F were moved to fresh wells prior to CV staining and imaging; as a result, no bacteria are present on the surfaces of the wells in these images.

### Proto-SLIPS Remain Physically Intact and Biologically Active after Exposure to Physical Insults

One aspect of potential practical utility not evaluated directly in the experiments above relates to the physical stability of these drug-patterned surfaces. Because these spots were deposited by solvent evaporation and are then exposed to infused lubricating oil, we considered the possibility that relatively weak interactions between the spots and the underlying porous substrate could permit them to dislodge or be removed prematurely. We note, at the outset, that we did not observe any drug spots to move or fall off during any of the routine handling involved in any of the experiments above. Nevertheless, to further evaluate physical stability, we subjected proto-SLIPS to several additional challenges, including overnight shaking on a shaker plate, incubation in an oven at 100 °C for 1 h, vortexing for 15 min, and at least 10 cycles of manual rubbing.

The results of these experiments are shown in the upper row images shown in Figure 6A. After all of these challenges, all gentamicin spots remained attached by visual inspection. Furthermore, subsequent ZOI assays, similar to those described above, confirmed their ability to release gentamicin and kill *S. aureus*. We also assessed the healing of these proto-SLIPS using the CV staining method outlined above; the results of those additional studies are shown in the lower row of images shown in Figure 6A. Finally, we also evaluated the initial shelf life stability of gentamicin-patterned proto-SLIPS by storing them ‘on the shelf’ for up to a month after preparation. During this period, samples were selected and assessed each week for their ability to kill bacteria when submerged in cultures of *S. aureus.* The results, shown in Figure 6B, reveal that proto-SLIPS are able to strongly kill bacteria after storage for up to 4 weeks. Overall, these results suggest that proto-SLIPs present a physically robust coating strategy to mitigate bacterial fouling and point toward practical applications for this methodology.

**Figure 6:**
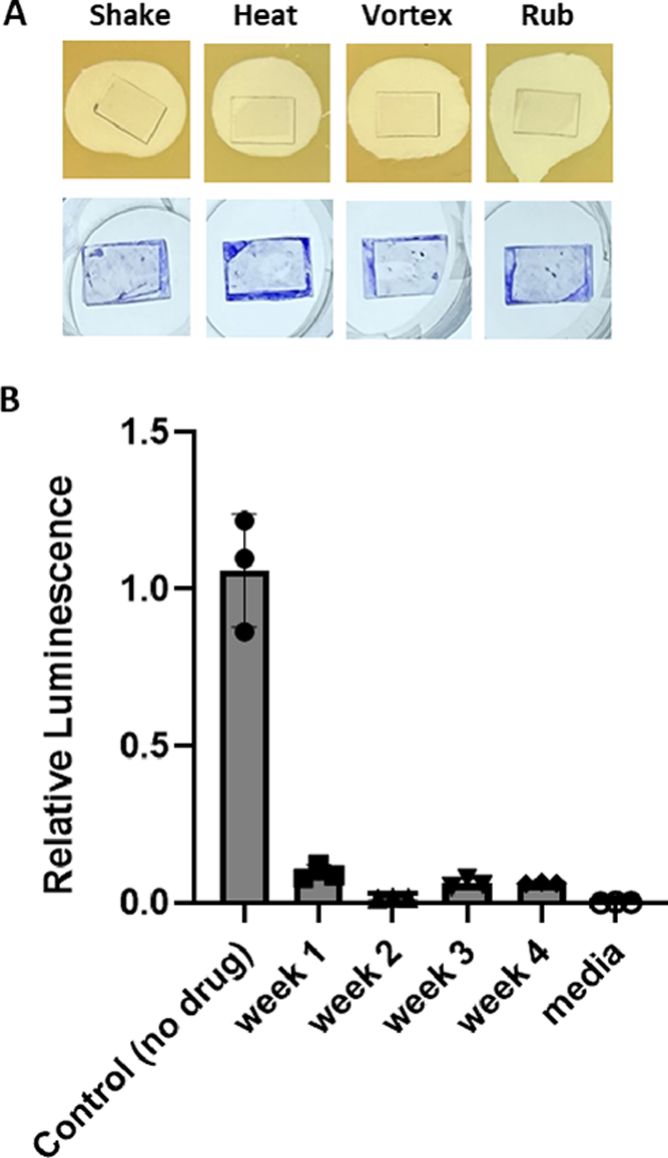
(A) The top row of images shows digital pictures showing the results of ZOI assays with *S. aureus* conducted using proto-SLIPS that were first subjected to various physical challenges (shaking, heating, vortexing, and manual rubbing; see text for additional details) prior to use. The proto-SLIPS used in these experiments were patterned with five drug spots created by evaporating 1 μL droplets of a 10 mg/mL aqueous gentamicin solution (analogous to samples in Figure 4C). The lower row of images in this panel shows representative top-down digital pictures of proto-SLIPS retrieved after the ZOI study mentioned above that were then incubated in liquid cultures of *S. aureus* for an additional 24 h before CV staining and subsequent imaging (see text). Retrieval of the samples from bacteria-seeded agar plates led to some movement of the PTFE membranes on the underlying glass slides. As noted and discussed further in the main text, areas of bare glass that were not covered by the proto-SLIPS were substantially fouled by biofilm in this liquid culture assay, evident in these samples as darker stained regions around the perimeters of the membranes. The proto-SLIPS themselves generally remained free of substantial biofouling under these conditions. (B) Results of quantitative well-based bioluminescence assays measuring *S. aureus* viability using proto-SLIPS that were stored on-the-shelf for different amounts of time prior to analysis. The graph shows the results of a triplicate of samples tested for each time point, with error bars representing the standard deviation of three measurements.

### Proto-SLIPS Patterned with Water-Soluble Antimicrobial Peptides Kill Fungal Pathogens

The overall design for proto-SLIPS is modular in nature and, in principle, can be adopted to design slippery, oil-infused surfaces that release a broad range of other highly water-soluble (or oil-insoluble) agents. To further explore the potential scope of this approach, we prepared proto-SLIPS patterned with dried spots of a model hydrophilic antimicrobial α,β-peptide having the structure G-^β^L-F-^β^K–I–I-^β^K–K-^β^I-A-^β^K–S–F-NH_2_. This synthetic peptide is highly water-soluble, insoluble in silicone oil, and is active against *Candida albicans*, an opportunistic fungal pathogen;^47^ this antifungal peptide is also charged [net charge of +5 at pH 7] and substantially more structurally complex than the small-molecule drug gentamicin used in the studies above, allowing us to further explore the scope of the proto-SLIPS approach. For these experiments, we used the same general procedure described above for the design of gentamicin-patterned surfaces to prepare proto-SLIPS with five peptide-patterned spots. In general, the dried peptide spots exhibited footprints larger than those observed for gentamicin spots because of an apparently stronger pinning force during the droplet evaporation process. The resulting peptide-patterned PTFE surfaces readily accepted the infusion of silicone oil to yield proto-SLIPS patterned with hydrophilic spots patterned with peptide.

Figure 7A-B shows representative results of an antifungal ZOI experiment performed using a conventional, unpatterned SLIPS surface (A) and a peptide-patterned proto-SLIPS surface (B). These experiments were performed in analogy to those described above for antibacterial experiments, with the exception that the agar plates on which the samples were placed were first inoculated with *C. albicans.* Inspection of Figure 7B reveals a clear zone present around the proto-SLIPS substrate consistent with the release and diffusion of the peptide from the surface. The images in Figure 7C–D show the results of an experiment in which a conventional SLIPS (C) and peptide-patterned proto-SLIPS substrate (D) were submerged directly into cultures of *C. albicans* for 24 h. These results are generally consistent with the results of immersion-based antibacterial assays described above, with the proto-SLIPS substrate able to, by visual inspection, reduce overall microbial load in solution and substantially reduce surface fouling relative to the conventional SLIPS substrate (as measured visually by examining turbidity and surface clarity, respectively). These peptide-patterned proto-SLIPS also recovered and healed into uniform, conventional SLIPS after the release of peptide, analogous to the surfaces described above.

**Figure 7:**
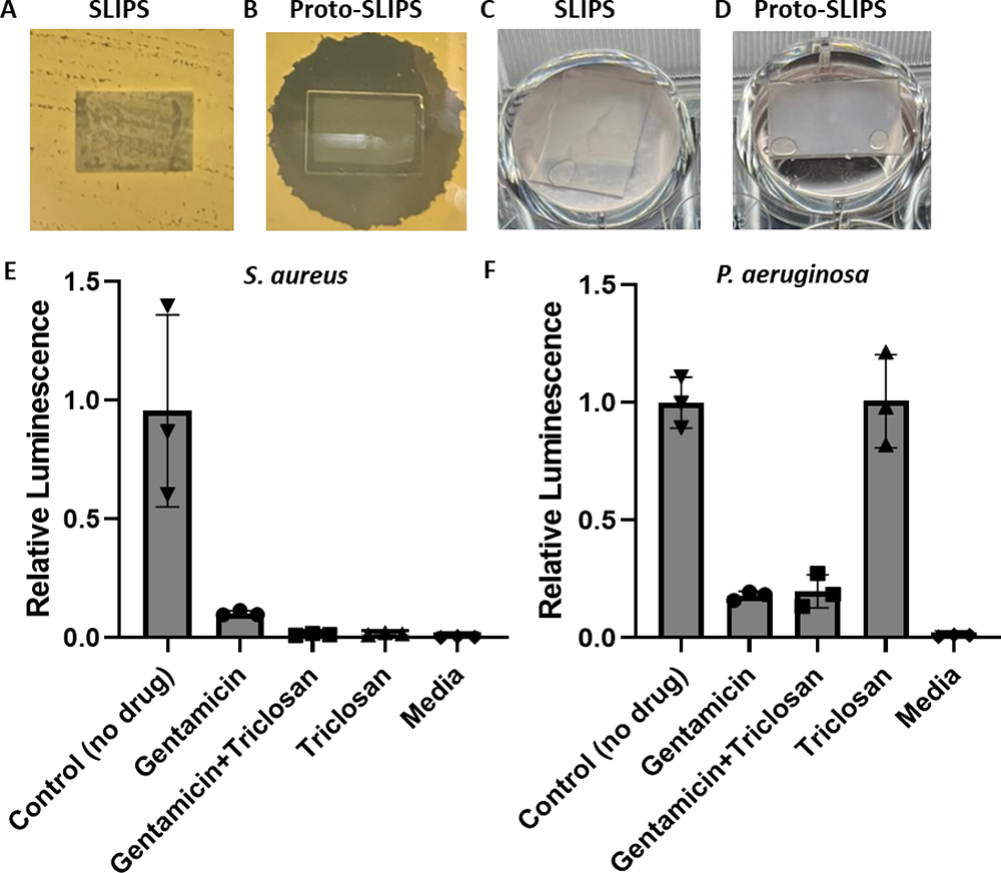
(A-B) Representative results of antifungal zone-of-inhibition (ZOI) experiments using a conventional unpatterned SLIPS surface (A) and an antifungal peptide-patterned proto-SLIPS surface (B) after 24 h of incubation on an agar plate inoculated with *C. albicans*. (C–D) Representative images showing the results of a submersion-based experiment in which conventional SLIPS surface (C) and a peptide-patterned proto-SLIPS surface (D) were incubated in *C. albicans* cultures; the results show a reduced microbial load and reduced surface fouling for the proto-SLIPS based on visual inspection of solution turbidity and surface clarity. (E-F) Plot showing relative levels of bioluminescence measured after the incubation of dual-release proto-SLIPS loaded with triclosan and/or gentamicin after 24 h of incubation with *S. aureus* (E) and *P. aeruginosa* (F). The plots show the averages of three independent cell viability assays, each with a triplicate of samples for all drug loading conditions. The error bars represent the standard deviation of the nine measurements.

### Fabrication of Proto-SLIPS Using Other Hydrophobic Porous Polymer Coatings

Additional experiments demonstrated that this drug-spotting approach could be applied to design drug-eluting proto-SLIPS using other hydrophobic porous polymer coatings that have been used in past studies for the design of SLIPS.^20,23,24,29,35^ Specifically, we conducted a series of studies to pattern gentamicin spots onto hydrophobic and nanoporous polymer multilayers fabricated by the reactive layer-by-layer assembly of polyethylene imine (PEI) and the amine-reactive polymer poly­(2-vinyl-4,4-dimethyl azlactone) (PVDMA), and then infused the coatings with silicone oil. We have shown previously that these porous PEI/PVDMA coatings can be used to fabricate conventional SLIPS on both simple and more topologically complex surfaces using a range of iterative immersion-, flow-, and spray-based methods.^48,49^ These gentamicin-patterned, PEI/PVDMA-based proto-SLIPS exhibited gentamicin-release and subsequent healing behaviors in aqueous environments that depended on the concentration of gentamicin deposited, with spots created using lower concentrations leading to faster recovery. Antibacterial assays also revealed substantial reductions in *S. aureus* growth, and the versatility of the layer-by-layer approach used to fabricate these coatings enabled the design of proto-SLIPS on the surfaces of flexible tubing, underscoring the potential to adopt this approach for the design of slippery, drug-eluting coatings on complex surfaces used to design catheters and other interventional devices. The results of these experiments are shown in Figure S3 and S4, and additional discussion of these results can be found in Note S1 of the Supporting Information.

### Leveraging the Oil Phase to Design ‘Dual-Release’ Proto-SLIPS that Release Both Water-Soluble and Oil-Soluble Agents

We were also interested in the potential to leverage this new proto-SLIPS design strategy for the release of highly water-soluble agents with other approaches that we reported previously for the release of oil-soluble agents from conventional SLIPS^20^ to provide potential ‘dual-release’ proto-SLIPS capable of hosting and releasing both oil-soluble *and* oil-insoluble agents. For example, we reported previously that triclosan, a hydrophobic and broad-spectrum antimicrobial drug that is at least partially soluble in silicone oil, can be loaded into conventional SLIPS and then slowly diffuse through the infused oil phase and partition into surrounding aqueous media to substantially reduce microbial loads.^20^ To determine whether this approach could be used in combination with the proto-SLIPS approach reported here, we first loaded triclosan into PTFE membranes using methods reported previously^20^ and then patterned dried gentamicin spots on the triclosan-loaded membranes using the methods introduced and discussed above. The overall evaporation behavior observed during and after the drying of the aqueous gentamicin droplets closely resembled those observed for the patterning of bare PTFE membranes (as in Figure 2), demonstrating that the pre-loading of triclosan into the PTFE membrane did not affect subsequent deposition of gentamicin. Proto-SLIPS containing only triclosan (no gentamicin) and only gentamicin (no triclosan) were also prepared as controls.

We evaluated these triclosan- and gentamicin-loaded ‘dual release’ proto-SLIPS using both ZOI and well-based immersion assays and two different species of bacteria: (i) *S. aureus* and (ii) *Pseudomonas aeruginosa.* These bacteria were selected to represent common Gram-positive and Gram-negative bacterial pathogens, respectively, and have different susceptibilities to triclosan and gentamicin (i.e., triclosan is active against *S. aureus* but is inactive in *P. aeruginosa*, while gentamicin is active against both *S. aureus* and *P. aeruginosa*).^50–53^ As shown in Figure 7E-F, SLIPS containing triclosan only were able to substantially reduce bacterial loads when submerged in cultures of *S. aureus* but did not reduce bacterial loads in cultures of *P. aeruginosa*. In these drug-loaded SLIPS, triclosan, which is oil-soluble, can diffuse or be transported through the infused oil phase and be released into the surrounding aqueous medium, yet it is, as expected, unable to kill *P. aeruginosa*. In contrast, proto-SLIPS patterned with gentamicin, which can kill both *S. aureus* and *P. aeruginosa*, are able to substantially reduce the growth of both species (Figure 7E-F). As shown in Figure S5A-B, these general responses to triclosan, gentamicin, and triclosan/gentamicin loaded PTFE surfaces were also observed in agar-based ZOI assays using these two bacterial species.

When combined, the results of these experiments demonstrate that both triclosan and gentamicin can be released, through different mechanisms, simultaneously and that these ‘dual release’ proto-SLIPS can also recover and transform to uniform conventional SLIPS in ways similar to those described above (Figure S5C). These results also further underscore the potential of this proto-SLIPS approach to serve as a general platform for the integration and release of multiple bioactive agents in ways that could help expand the antimicrobial and/or therapeutic reach of these antifouling coatings. We note that, while the experiment described here was designed specifically for demonstration purposes (because proto-SLIPS that release gentamicin alone can kill both *S. aureus* and *P. aeruginosa*) the results above demonstrate, via proof-of-concept, that the proto-SLIPS approach itself can be used to add additional value to triclosan-loaded SLIPS, which are unable to, themselves, kill *P. aeruginosa*. Overall, we expect that these two approaches can be used to design SLIPS that release strategic combinations of oil-soluble and/or highly water-soluble (oil-insoluble) agents to produce antifouling coatings that are active in or against a broad range of microbial and mammalian cell types (or that may have other desirable effects).

### Design Strategies for the Generation of Proto-SLIPS that Promote Sustained Release

The approach to the design of proto-SLIPS described above takes advantage of the general insolubility of highly water-soluble agents in infused oil phases to design slippery surfaces containing spots of drug that dissolve immediately upon introduction to aqueous solution; depending on the drug loading and solubility, patterned spots can fully dissolve in less than a second or within a few minutes when immersed in water. This approach yields surfaces that rapidly release or ‘dump’ their active agent and then quickly self-heal, and is, thereby, useful for affecting immediate reductions in microbial load in the vicinity of what will be (or quickly become) an otherwise inherently antibiofouling surface. In this final section, we report two alternate designs for the patterning of drug spots that provide useful ways to prolong or sustain the release of highly water-soluble agents.

In the first approach, we explored the potential utility of depositing a hydrophobic degradable polymer to physically cover patterned gentamicin spots and slow down or delay the dissolution and dissemination of gentamicin. This general concept is illustrated schematically in Figure 8A. To demonstrate proof of concept, we first patterned spots of gentamicin on a PTFE membrane (using 5 μL droplets of a 10 mg/mL gentamicin solution and methods described above) and then deposited a small volume (5 μL) of a solution of the hydrolytically degradable polymer poly­(ε-caprolactone) (PCL, 3 wt % in dichloromethane; depicted in green in Figure 8A) on top of the dried gentamicin spots (depicted in red). After the polymer solution evaporated, the PTFE membranes were infused with silicone oil. These proto-SLIPS were then used in a multiple-challenge bacterial immersion assay in which the surfaces were exposed to a first culture of *S. aureus* for an initial period of 24 h, removed and rinsed, and then transferred to a second culture of fresh *S. aureus* for an additional 24 h. As shown in Figure 8B, gentamicin-patterned proto-SLIPS (dark gray bars) without PCL caps were able to strongly attenuate bacterial load during the initial 24-h period but were unable to substantially kill bacteria during the second 24-h period, consistent with the rapid release observed in experiments in the sections above. In contrast, proto-SLIPS with PCL-capped gentamicin spots were able to substantially reduce bacterial load during the first 24-h period *and* significantly kill bacteria during the second 24-h cycle (albeit at a level that was reduced compared to the level of activity in the first 24-h period). This result is consistent with a slower release of gentamicin from the PCL-capped spots over a periods of at least 24 h, relative to uncapped spots, and the overall physical picture presented in Figure 8A. Additional experiments will be required to more fully understand the structures of the PCL-capped spots, the mechanisms by which they can act to sustain release, the extent to which self-healing can be achieved, and the parameters that can be used to further tune or optimize them in the context of specific applications. In the context of this current study, we interpret this result to hint at a range of polymer-based spot-coating strategies that could be used to further attenuate the drug release profiles proto-SLIPS.

**Figure 8:**
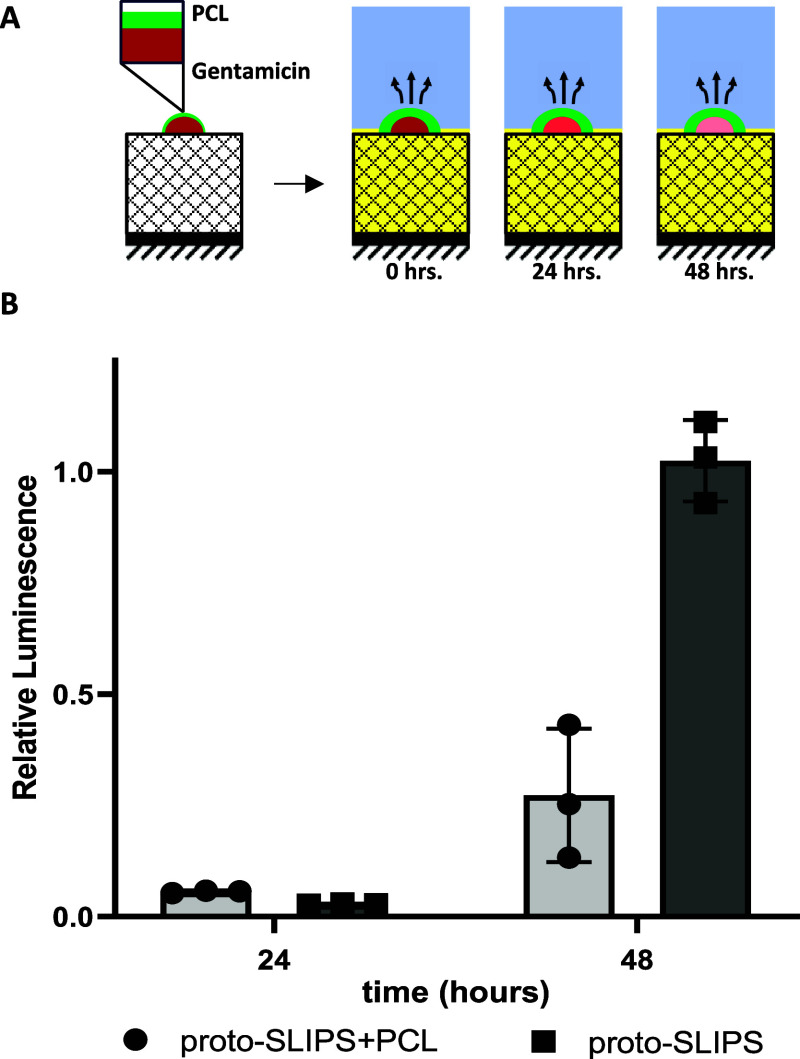
(A) Schematic representation showing a gentamicin spot (red) covered by a hydrophobic hydrolytically degradable polymer, poly­(ε-caprolactone) (PCL, shown in green) patterned on a porous PTFE membrane (white). These patterned features remain after infusion of the PTFE membrane with silicone oil (yellow), providing potential means to sustain or delay the release of gentamicin. (B) Plot of relative bioluminescence as a function of time for proto-SLIPS patterned with gentamicin spots only (dark gray bars) and proto-SLIPS patterned with gentamicin spots covered with PCL (light gray bars). Both samples were submerged in a first culture of *S. aureus* for 24 h and then removed and incubated in a second fresh bacterial culture for an additional 24 h. Bacterial viability was quantified using a bioluminescence-based assay; values were corrected relative to bacteria controls; lower values indicate fewer live bacteria. The plot shows the result of a triplicate of samples tested for each condition, with error bars representing the standard deviation of the three measurements.

In a second and complementary approach, we explored strategies to achieve the sustained release of active agents by creating proto-SLIPS containing patterned spots containing degradable polymer microparticles. This general approach is illustrated schematically in Figure 9A. To explore feasibility and demonstrate proof of concept, we deposited aqueous droplets containing polymer microparticles fabricated using the hydrolytically degradable polymer poly­(lactide-*co*-glycolide) (PLG) onto PTFE membranes using the same general approach described above for gentamicin-patterned proto-SLIPS. For these experiments, we formulated the aqueous droplets containing PLG microparticles (depicted in purple in Figure 9A) to also contain a small amount of poly­(ethylene glycol) (PEG, 33 wt % in water; depicted in orange) as a binder that could (i) promote initial adhesion of the microparticles after evaporation of water but (ii) dissolve rapidly upon re-exposure to bulk water and, thereby, (iii) promote the rapid release and dissemination of the microparticles and subsequent self-healing (Figure 9A).

**Figure 9:**
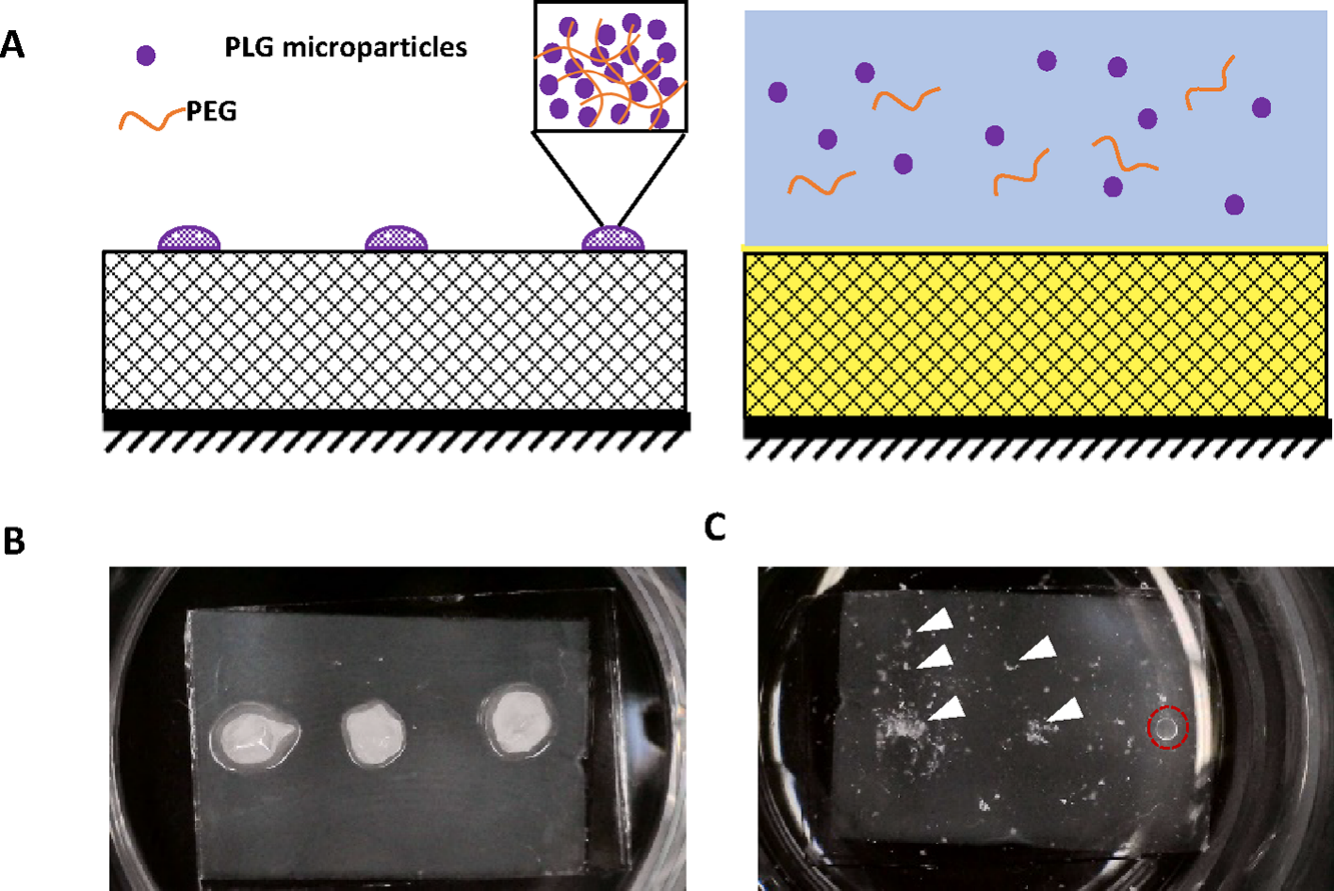
(A) Schematic illustration showing a porous PTFE membrane (white) patterned with degradable polymer microparticles (shown in purple) created by the deposition and evaporation of an aqueous droplet containing microparticles and dissolved PEG (orange) as a binding agent. Subsequent infusion of the PTFE membrane with silicon oil (yellow) yields proto-SLIPS that can rapidly release degradable microparticles upon introduction to aqueous environments (blue) and then undergo processes of self-healing and repair to transform into uniform slippery surfaces. The polymeric microparticle assemblies can disseminate and be used to sustain the release of other agents in the vicinity of the self-repaired slippery surface. (B) Representative image of a proof-of-concept PLG microparticle-patterned proto-SLIPS surface after infusion with silicone oil; the white patches are microparticle assemblies. (C) Image showing the same proto-SLIPS surface after exposure to water for 15 min and the subsequent rapid release and dissemination of microparticles into the surrounding environment. White arrowheads point to examples of large clusters of microparticles that have been released from the surface, as evident by their mobility when the media was gently shaken; the red circle marks the location of an air bubble trapped between the PTFE membrane and the supporting glass substrate (underneath the SLIPS coating itself).

Figure 9B shows an image of a model PLG microparticle-patterned proto-SLIPS surface after infusion with silicone oil. This coating was patterned with three microparticle spots, all of which remained bound to the surface after infusion with oil. Figure 9C shows an image of this same proto-SLIPS surface after placing it in water for 15 min. Inspection of this image reveals that the microparticles were released and disseminated rapidly into the surrounding environment under these conditions. Following rapid release, these surfaces recovered and transformed to uniform SLIPS, as determined by characterizing the behaviors of sliding water droplets and in ways similar to other examples described above (e.g., 10 μL water droplets were able to slide freely at an angle of 10° with no pinning). Although we used ‘empty’ (unloaded) PLG microparticles for demonstration purposes in this proof-of-concept experiment, we anticipate that this approach could also be used with myriad formulations of degradable PLG microparticles encapsulating a wide range of small-molecule and macromolecular cargo (e.g., proteins, peptides, nucleic acids, etc.), including both hydrophilic and/or hydrophobic agents. In this way, this strategy provides access to a proto-SLIPS platform that could promote the rapid release of well-known controlled release drug delivery formulations^33^ that can persist in areas surrounding an inherently antifouling and self-repaired coating and, thereby, sustain the release of active agents in the vicinity of these surfaces for additional hours, days, or months.

## Summary and Conclusions

We have reported an approach to enhance the antibiofouling properties of slippery liquid-infused surfaces (SLIPS) using a drug-patterning ‘proto-SLIPS’ approach that enables the rapid release of defined quantities of highly water-soluble agents. This approach addresses a general limitation of many conventional SLIPS-based coatings, which are often very effective at preventing surface contamination by microorganisms but do not possess the means to kill nonadherent microorganisms or reduce cell loads in surrounding environments. Our results demonstrate that proto-SLIPS can effectively release patterned, oil-resistant spots of gentamicin, a clinically useful small-molecule antibiotic, rapidly into surrounding aqueous environments and then undergo processes that lead to rapid self-healing and transformation to uniform slippery surfaces typical of conventional SLIPS coatings. Our results also show that control over the rates and extents of drug release and self-healing can be tuned through variations in loading volume, drug concentration, and modes of evaporative drying during the preparation of the patterned drug spots. The results of microbiological studies demonstrate that these gentamicin-patterned proto-SLIPS can effectively kill the common bacterial pathogen *S. aureus* growing on the surfaces of agar or in liquid culture medium and undergo self-healing, after drug release, to a degree that leads to otherwise continuous and conventional SLIPS coatings that are able to substantially prevent further bacterial surface fouling.

This proto-SLIPS approach is modular in nature; we anticipate that this drug-spotting and self-healing approach should be useful for the preparation of proto-SLIPS surfaces that can release a wide variety of different highly water-soluble and/or oil-soluble agents. In support of this view, our results demonstrate that proto-SLIPS patterned with a charged and hydrophilic synthetic antifungal peptide—an active antimicrobial agent with a structure and physical properties that are very different from those of gentamicin—can be used to kill the opportunistic fungal pathogen *C. albicans*. Our results also demonstrate that the oil phases in proto-SLIPS can be leveraged to design coatings that promote the simultaneous release of gentamicin (via dissolution of patterned drug spots) and the broad-spectrum antimicrobial triclosan (via slower and more sustained elution from the infused oil phase) and thereby deliver combinations of drugs that are active against populations of multiple bacterial species. Finally, this basic proto-SLIPS approach can be combined with a variety of tools and approaches developed within the drug delivery community to provide new pathways to sustain and control the release of patterned agents. While the majority of the proof-of-concept studies reported here were performed using proto-SLIPS prepared using model porous PTFE substrates, our results also show that this approach can also be extended to the use of other porous polymer coatings commonly used to prepare conventional SLIPS on a variety of curved and complex real-world surfaces (e.g., flexible polymer tubing). Overall, this proto-SLIPS strategy presents a new, useful, and modular approach to the design of drug-eluting SLIPS coatings that has the potential to improve the antibiofouling behaviors of these materials and, with further development, open the door to new applications of slippery liquid-infused coatings in healthcare and other applied contexts.

## Supplementary Material





## Notes

Additional information including images, plots, and text characterizing the structures, physicochemical properties, and antibiofouling behaviors of proto-SLIPS coatings (PDF)
Videos showing dynamic behaviors of proto-SLIPS surfaces (MP4)
