# Supplementary material for: Proto-SLIPS: Slippery Liquid-Infused Surfaces that Release Highly Water-Soluble Agents
Source: ACS Appl Mater Interfaces. 2025 Sep 9;17(38):54166–80. doi: 10.1021/acsami.5c09975 (PMC12425458; doi:10.1021/acsami.5c09975)
Supplement: Supplementary file 1 [file am5c09975_si_001.pdf]

SUPPORTING INFORMATION

Proto-SLIPS: Slippery Liquid-Infused Surfaces that Release Highly Water-Soluble Agents

Fengrui Wang,[1,†] Jordan T. York,[1,†] Takuma N. Kawamura,[2] Douglas H. Chang,[2] Sean P. Palecek,[2,*] Helen E. Blackwell,[1,*] and David M. Lynn[1,2,*]

[1]Department of Chemistry, University of Wisconsin–Madison, 1101 University Ave., Madison, WI 53706, USA; [2]Department of Chemical and Biological Engineering, University of Wisconsin–Madison, 1415 Engineering Dr., Madison, WI 53706, USA; [†]Equally contributing author; Email: sppalecek@wisc.edu (S.P.P), blackwell@chem.wisc.edu (H.E.B.),dlynn@engr.wisc.edu (D.M.L.)

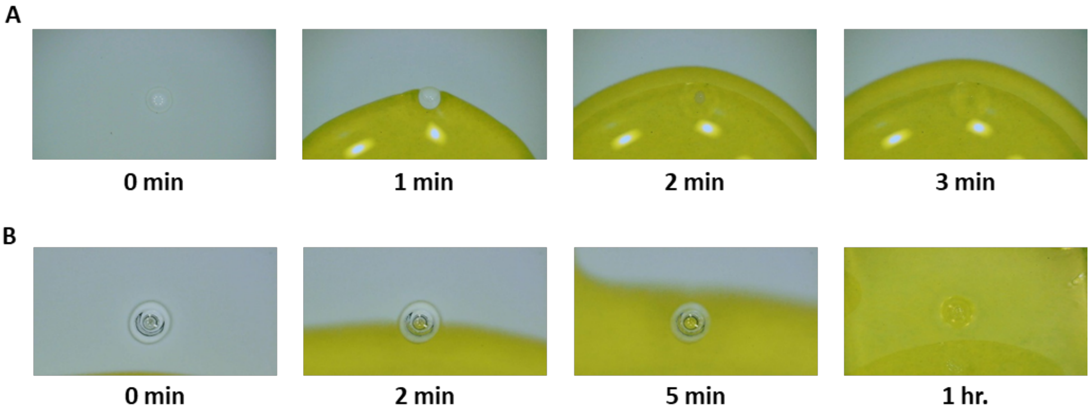

Figure S1. Representative top-down time-course images characterizing the infusion of silicone oil into proto-SLIPS. (A) Images showing the infusion of silicone oil at different timepoints for proto-SLIPS created by the evaporation of 5 µL droplets of a 10 mg/mL gentamicin solution. The glass-mounted PTFE membrane patterned with gentamicin was placed on a yellow background prior to infusion, and as oil starts to infuse into the membrane the opaque membrane became transparent, revealing the yellow background behind it and providing a visual indicator of regions that are infused and regions that are not. For drug spots created using 10 mg/mL gentamicin solutions (A), the oil infuses in areas of the membrane surrounding the spots before it infuses into areas of the membrane directly under the spots, but subsequent infusion in areas under the spots occurs rapidly (e.g., within 2-3 minutes). (B) Images showing the infusion of oil for proto-SLIPS created by evaporating 10 µL droplets of a 100 mg/mL gentamicin solution. In this case, the area under the center region of the drug spot containing the large air pocket (the air pocket can be observed in the image acquired at 0 minutes) became transparent before the rest of the surrounding area covered by the drug spot. This surrounding area is visible as an opaque white concentric ring around the center of the spot, which is visible as yellow, in the image acquired at 5 minutes). This remaining area under the drug spots required longer times to become fully transparent, as indicated by the onset of full transparency shown at the 1-hour timepoint.

Table S1. Measured dimensions of the footprints of gentamicin spots created by the evaporation of aqueous solutions of gentamicin as a function of droplet volume and concentration and the associated times required for proto-SLIPS patterned under these conditions to regain slipperiness (defined as the de-pinning time) such that water droplets could slide across patterned areas of the surface without impediment (see text). All measurements were made in triplicate and are shown as averages with standard deviation.

	Drug solution concentration			
	10 mg/mL		100 mg/mL	
Drug dot volume	Footprint (mm)	De-pinning time (s)	Footprint (mm)	De-pinning time (s)
0.1 μL	0.3±0.15	<1	0.8±0.2	3.7±1.1
5 μL	0.6±0.5	15.3±7.7	1.3±3.1	91.7±9.6
50 μL	14.0±1.5	127±6.8	55.3±5.0	266±29.1

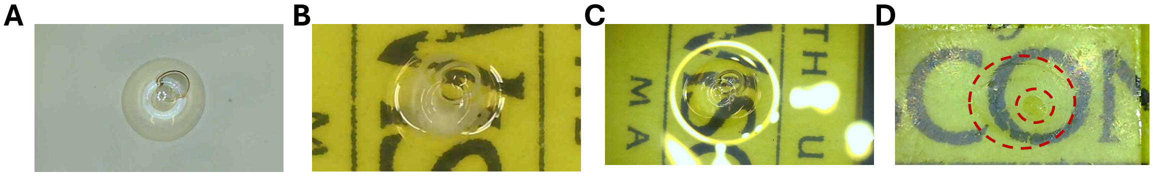

Before oil infusion | Oil infusion 1 hr. | Oil infusion 24 hr. | After drug release

Figure S2. (A-C) Top-down images showing the oil infusion process at different timepoints for proto-SLIPS created by the evaporation of a 100 µL droplet of a 100 mg/mL gentamicin solution. The glass-mounted PTFE membrane patterned with gentamicin was placed on a yellow background with black printing prior to infusion, and as oil started to infuse into the membrane the opaque membrane (seen as white in panel A) became transparent, revealing the yellow background behind it and providing a visual indicator of regions that are infused and regions that are not. After 1 hour of oil infusion, the area surrounding the drug spot and the area beneath the air pocket trapped within the drug spot were transparent, suggesting that these areas were being infused first (this is generally consistent with the discussion above and results shown in Figure S1, although the overall process is slower in the substantially larger spots used here). After 24 hours, the entire area covered by the drug spot was transparent, consistent with complete oil infusion. (D) Image showing the area of the proto-SLIPS shown in panels A-C after exposing it to water to dissolve the gentamicin and then subsequently removing the water. Glossy areas indicating areas of excess oil (highlighted by the red dashed circles) were observed in the location where gentamicin was patterned and, in particular, in locations corresponding roughly to areas that were infused first (e.g., the edge of the spot and the air pocket in the center of the spot, as indicated by the red dashed circles), suggesting that these regions contain excess oil that is available for transport into other locations of the membrane to promote healing and self-repair.

Note S1: Fabrication of Proto-SLIPS Using Hydrophobic Polymer Multilayers

We also sought to explore the extent to which the drug-patterning approach reported using PRFE membranes as substrates could be used to design proto-SLIPS using other types of polymer coatings used in past studies for the design of conventional SLIPS. For these studies, we used a class of hydrophobic and nanoporous polymer coatings fabricated by the reactive layer-by-layer assembly of polyethylene imine (PEI) and the amine-reactive azlactone-containing polymer poly(2-vinyl-4,4-dimethyl azlactone) (PVDMA).[1-6] We have shown previously that these PEI/PVDMA coatings can be used to fabricate conventional SLIPS on both simple and more topologically complex surfaces using a range of iterative immersion-, flow-, and spray-based methods (e.g., as shown in Figure S3A). [4, 7-9]

We patterned the surfaces of PEI/PVDMA coatings fabricated on planar glass substrates using small aqueous droplets of gentamicin and the evaporation-based methods described in the main text for the design of PTFE-based proto-SLIPS. Owing to the different and more complex morphology of these coatings as compared to porous PTFE membranes, we observed variations in droplet behaviors during the evaporation process that affected dried gentamicin spot sizes. Notably, gentamicin spots created using aqueous solutions with lower gentamicin concentrations exhibited evaporation patterns dominated by CCR modes, while droplets with higher concentrations of gentamicin led to spots with footprints that were larger than those of the original deposited water droplets (see Figure S4 for details). All gentamicin-patterned PEI/PVDMA coatings could be successfully infused with silicon oil, but we also observed differences in subsequent healing and recovery processes after these proto-SLIPS were placed in aqueous environments. In general, proto-SLIPS created using lower concentrations of gentamicin solutions (10 mg/mL) displayed rapid healing in times ranging from five minutes to one hour, depending on

the volumes used to create the spot. Conversely, drug spots created using higher concentrations of gentamicin solution (100 mg/mL) exhibited longer and more irregular healing times that ranged from hours to several days.

Figure S3C shows the results of immersion-based antimicrobial assays 24 hours after the immersion of these PEI/PVDMA-based proto-SLIPS, patterned by the evaporation of five 1 μL droplets of 10 mg/mL, in cultures of *S. aureus.* These results reveal proto-SLIPS fabricated by both spray-based methods and immersion-based methods to substantially reduce bacterial load relative to conventional PEI/PVDMA-based SLIPS. Additional ZOI assays conducted using these surfaces revealed results similar to those shown above for PTFE-based proto-SLIPS. Finally, to demonstrate the potential for this layer-by-layer coating approach to enable the fabrication of drug-eluting proto-SLIPS on more complex surfaces, we coated the outer surface of a flexible plastic tube similar to that used to design vascular catheters. The image shown in Figure S3B shows a segment of proto-SLIPS-coated tubing patterned with small gentamicin spots; as shown in Figure S3C, this proto-SLIPS-coated tubing was also able to substantially kill bacteria when incubated in cultures of *S. aureus.*

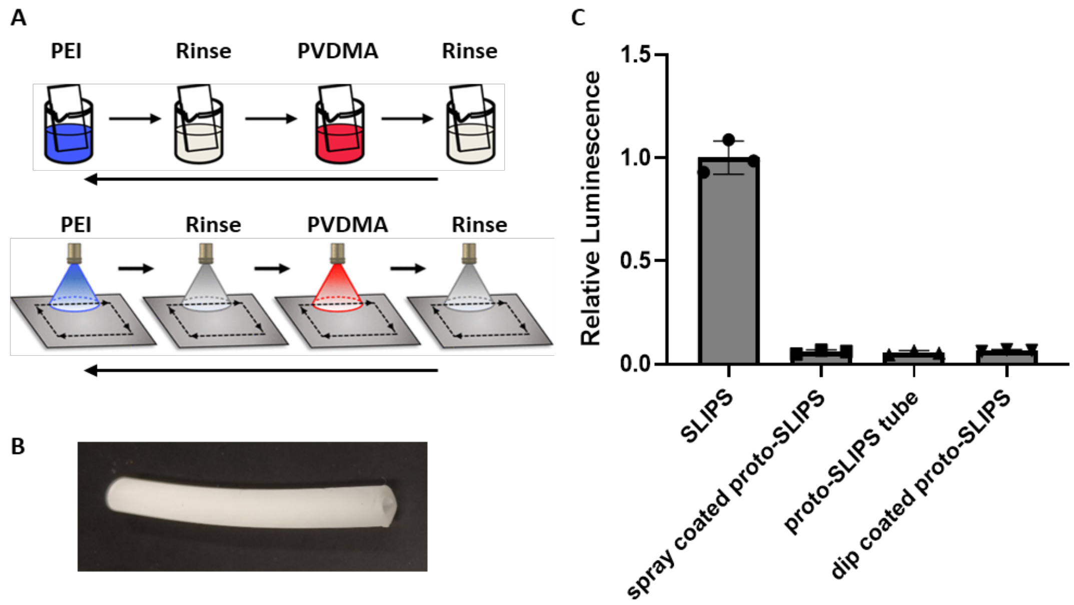

Figure S3. (A) Schematic illustration of the iterative processes used for the fabrication of PEI/PVDMA-based polymer multilayers. Substrates were exposed sequentially to solutions of PEI and PVDMA using iterative immersion (top) or sequential spraying (bottom) methods, yielding porous hydrophobic coatings that could be infused with oil to form SLIPS. (B) Representative image of a segment of a flexible plastic tube coated with a gentamicin-patterned PEI/PVDMA-based proto-SLIPS coating. (C) Plot showing the results of antibacterial assays, expressed as relative levels of bioluminescence in cultures of S. aureus, 24 hours after the incubation of conventional PEI/PVDMA-based SLIPS and gentamicin-patterned PEI/PVDMA-based proto-SLIPS fabricated using immersion- or spray-based methods. All coatings used in these studies were fabricated on planar glass substrates, with the exception of the example coated on flexible plastic tubing, as noted in panel C and shown in panel B. The plot shows the result of a triplicate of samples tested for each condition, with error bars representing the standard deviation of the three measurements.

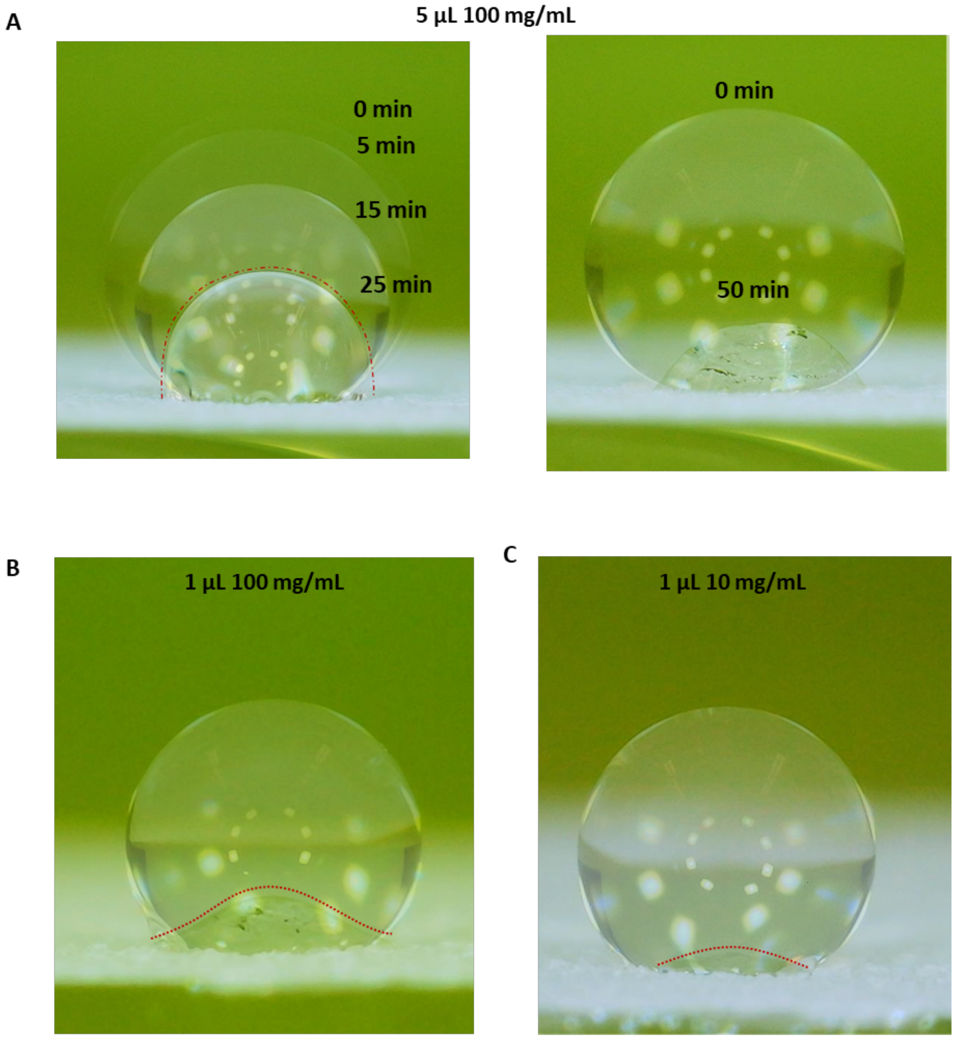

Figure S4. Analysis of droplet behavior observed during the evaporation of gentamicin droplets loaded on PEI/PVDMA-based porous polymer coatings. (A) The left panel shows superimposed time-lapse photos of the evaporation process of a 5 µL droplet of a 100 mg/mL gentamicin solution on a PEI/PVDMA coating. Between 15-25 mins, a significant increase in droplet footprint was observed. The right panel shows superimposed pictures of the same droplet at time 0 and after 50 minutes of evaporation (the final dried drug spot is outlined with a dotted red line to guide the eye). The final drug spot had a noticeably larger footprint than that of the originally deposited droplet. (B-C) Superimposed images showing initially deposited droplets and their final evaporated drug spots (the latter are outlined with a dotted red line to guide the eye). The image in (B) shows the evaporation of a 1 µL droplet of a 100 mg/mL gentamicin solution. The evaporated drug spot had a larger footprint than that of the original droplet deposited. The image in (C) shows an example of the evaporation of a 1 µL droplet of a 10 mg/mL gentamicin solution. The evaporated drug spot had a footprint that was similar to that of the original deposited droplet.

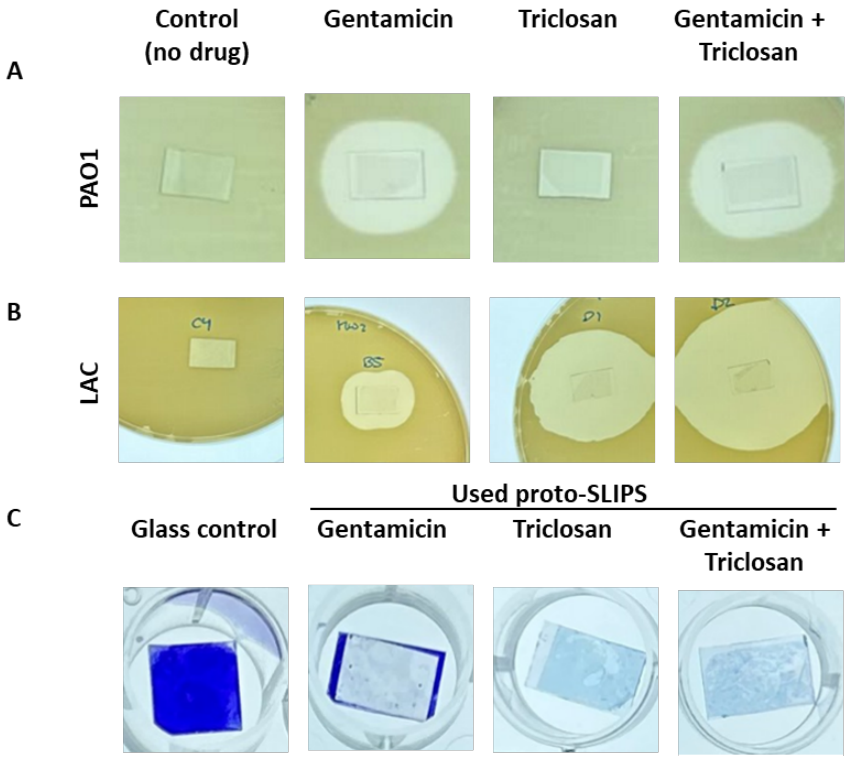

Figure S5. Examples of dual-release proto-SLIPS evaluated using ZOI assays and analysis of subsequent healing processes using a CV staining assay. (A-B) Representative results of ZOI experiments using the PAO1 (wild-type) strain of *P. aeruginosa* (A) and the LAC strain of *S. aureus* (B) using a conventional un-patterned SLIPS surface, a proto-SLIPS loaded with gentamicin only, an un-patterned SLIPS loaded with triclosan only, and a proto-SLIPS loaded with both gentamicin and triclosan (see main text for additional details and discussion). (A) Representative images after 24-hour incubation of samples face-down on agar plates inoculated with PAO1. Clearing zones were observed only in samples containing gentamicin; because *P. aeruginosa* is resistant to triclosan, no clearing zone is observed in the triclosan-only case. (B) Representative images after 24-hour incubation of samples face-down on agar plates inoculated with LAC. Clearing zones were observed in all samples containing gentamicin and/or triclosan. (C) Images showing the results of CV staining biofilm analysis of representative samples used in the experiments in panels A and B along with a bare glass control after an additional 24 hours of incubation in liquid cultures of *S. aureus* (MW2 strain). The glass substrate is observed to have substantial biofilm growth. The area of the supporting glass slide covered by the gentamicin-loaded proto-SLIPS (second from left) remains colorless, consistent with a substantial reduction in biofilm growth, while the surrounding and exposed areas of the glass substrate show significant staining. Samples loaded with triclosan (with or without gentamicin; the two right-most images) were still able to release triclosan during this secondary incubation step (release of gentamicin occurs within minutes, whereas release of triclosan can occur for extended periods). As a result, both the surface of the proto-SLIPS and the exposed areas of glass surrounding the membranes remain relatively free of stain.

Supporting Video

Video S1. Representative video showing a side-on view of part of the evaporation process of a 5 μL droplet of a 10 mg/mL gentamicin solution on a PTFE membrane. As evaporation takes place, air pockets start to appear and then later disappear as they escape from the droplet as the droplet footprint retracts.

Supporting Information References

1. Manna, U.; Broderick, A. H.; Lynn, D. M. Chemical Patterning and Physical Refinement of Reactive Superhydrophobic Surfaces. *Adv. Mater.* **2012**, *24* (31), 4291-4195.

2. Manna, U.; Kratochvil, M. J.; Lynn, D. M. Superhydrophobic Polymer Multilayers that Promote the Extended, Long-Term Release of Embedded Water-Soluble Agents. *Adv. Mater.* **2013**, *25* (44), 6405-6409.

3. Manna, U.; Lynn, D. M. Patterning and Impregnation of Superhydrophobic Surfaces Using Aqueous Solutions. *ACS Appl. Mater. Interfaces* **2013**, *5* (16), 7731-7736.

4. Manna, U.; Lynn, D. M. Fabrication of Liquid-Infused Surfaces Using Reactive Polymer Multilayers: Principles for Manipulating the Behaviors and Mobilities of Aqueous Fluids on Slippery Liquid Interfaces. *Advanced Materials* **2015**, *27* (19), 3007-3012.

5. Manna, U.; Lynn, D. M. Synthetic Surfaces with Robust and Tunable Underwater Superoleophobicity. *Adv. Funct. Mater.* **2015**, *25* (11), 1672-1681.

6. Manna, U.; Raman, N.; Welsh, M. A.; Zayas-Gonzalez, Y. M.; Blackwell, H. E.; Palecek, S. P.; Lynn, D. M. Slippery Liquid-Infused Porous Surfaces that Prevent Microbial Surface Fouling and Kill Non-Adherent Pathogens in Surrounding Media: A Controlled Release Approach. *Adv. Funct. Mater.* **2016**, *26* (21), 3599-3611.

7. Agarwal, H.; Breining, W. M.; Sanchez-Velazquez, G.; Lynn, D. M. Reactive Multilayers and Coatings Fabricated by Spray Assembly: Influence of Polymer Structure and Process Parameters on Multi-Scale Structure and Interfacial Properties. *Chem. Mater.* **2022**, *34*, 1245–1258.

8. Agarwal, H.; Breining, W. M.; Lynn, D. M. Continuous Fabrication of Slippery Liquid-Infused Coatings on Rolls of Flexible Materials. *ACS Appl. Polym. Mater.* **2022**, *4*, 787–795.

9. Agarwal, H.; Nyffeler, K. E.; Blackwell, H. E.; Lynn, D. M. Fabrication of Slippery Liquid-Infused Coatings in Flexible Narrow-Bore Tubing. *ACS Appl. Mater. Inter.* **2021**, *13*, 55621-55632.
